# Monocyte/Macrophage‐Mediated Transport of Dual‐Drug ZIF Nanoplatforms Synergized with Programmed Cell Death Protein‐1 Inhibitor Against Microsatellite‐Stable Colorectal Cancer

**DOI:** 10.1002/advs.202405886

**Published:** 2024-08-05

**Authors:** Xietao Ye, Yuping Liu, Liangyin Wei, Yeyang Sun, Xiaoran Zhang, Hong Wang, Hong Guo, Xiaoying Qin, Xiaoqi Li, Ding Qu, Jiege Huo, Yan Chen

**Affiliations:** ^1^ Affiliated Hospital of Integrated Traditional Chinese and Western Medicine Nanjing University of Chinese Medicine Nanjing 210028 China; ^2^ Multi‐component of Traditional Chinese Medicine and Microecology Research Center Jiangsu Province Academy of Traditional Chinese Medicine Nanjing 210028 China; ^3^ Jiangsu Clinical Innovation Center of Digestive Cancer of Traditional Chinese Medicine Nanjing 210028 China

**Keywords:** antigen transport, combination therapy, MHC‐I, monocyte/macrophage transport, PD‐1 inhibitors

## Abstract

Microsatellite‐stable colorectal cancer (MSS‐CRC) exhibits resistance to programmed cell death protein‐1 (PD‐1) therapy. Improving the infiltration and tumor recognition of cytotoxic T‐lymphocytes (CTLs) is a promising strategy, but it encounters huge challenges from drug delivery and mechanisms aspects. Here, a zeolitic imidazolate framework (ZIF) coated with apoptotic body membranes derived from MSS‐CRC cells is engineered for the co‐delivery of ginsenoside Rg1 (Rg1) and atractylenolide‐I (Att) to MSS‐CRC, named as Ab@Rg1/Att‐ZIF. This system is selectively engulfed by Ly‐6C^+^ monocytes during blood circulation and utilizes a “hitchhiking” mechanism to migrate toward the core of MSS‐CRC. Ab@Rg1/Att‐ZIF undergoes rapid disassembly in the tumor, released Rg1 promotes the processing and transportation of tumor antigens in dendritic cells (DCs), enhancing their maturation. Meanwhile, Att enhances the activity of the 26S proteasome complex in tumor cells, leading to increased expression of major histocompatibility complex class‐I (MHC‐I). These coordinated actions enhance the infiltration and recognition of CTLs in the center of MSS‐CRC, significantly improving the tumor inhibition of PD‐1 treatment from ≈5% to ≈69%. This innovative design, involving inflammation‐guided precise drug co‐delivery and a rational combination, achieves synergistic engineering of the tumor microenvironment, providing a novel strategy for successful PD‐1 treatment of MSS‐CRC.

## Graphical abstract

A nanoplatform for monocyte/macrophage‐mediated transport in vivo to enhance the therapeutic effects of αPD‐1 against MSS CRC. Ab@Rg1/Att‐ZIF is transported to the tumor center, which regulates maturation in DCs and MHC‐I expression in tumor cells, respectively. It improves the therapeutic efficacy of αPD‐1 in MSS CRC by promoting CTLs infiltration into the tumor and attacking tumor cells accurately.

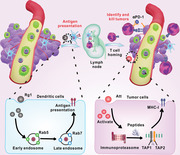

## 1 Introduction

Colorectal cancer (CRC) is the third most common malignant tumor worldwide, with its mortality rate ranking second among all malignant tumors.^[^ ^1^ ^]^ Most patients with CRC are at an advanced stage at the time of treatment, and the efficacy of chemotherapy^[^ ^2^ ^]^ and targeted therapy^[^ ^3^ ^]^ is limited. In recent years, immune checkpoint blockade therapy represented by programmed cell death protein 1 (PD‐1) inhibitors has been used to treat CRC. However, the efficacy of PD‐1 inhibitors varies across different phenotypes of CRC.^[^ ^4^ ^]^ The clinical classification of CRC is mainly based on microsatellite instability (MSI‐H) and microsatellite stability (MSS).^[^ ^5^ ^]^ PD‐1 inhibitors have demonstrated good therapeutic efficacy in 15% of all CRC patients exhibit MSI‐H (objective response rate, 53%).^[^ ^4^ ^]^ However, the remaining 85% of patients with MSS CRC have almost no response to PD‐1 inhibitors.^[^ ^6^ ^]^

The anti‐tumor function of PD‐1 inhibitors primarily relies on the infiltration number of cytotoxic T‐lymphocytes (CTLs) and their ability to recognize and kill tumor cells.^[^ ^7^ ^]^ On the one hand, infiltration of CTLs into tumor tissues requires dendritic cells (DCs) efficiently present tumor antigens to T cells in lymph nodes to recruit CTLs to tumor sites.^[^ ^8^ ^]^ DCs engulf tumor antigens and are processed for presentation to T cells with endosomal transport,^[^ ^9^ ^]^ but antigen processing and presentation by DCs in the tumor microenvironment is impaired,^[^ ^10^ ^]^ thereby reducing CTLs infiltration in MSS CRC tissues.^[^ ^11^ ^]^ On the other hand, the ability of CTLs to specifically recognize tumor cells is necessary for the anti‐tumor activity of PD‐1 inhibitors.^[^ ^12^ ^]^ CTLs need to bind to antigens presented by major histocompatibility complex class I (MHC‐I) molecules on the surface of tumor cells to effectively recognize and kill tumor cells.^[^ ^13^ ^]^ MSS CRC cells inhibit the activity of the 26S immunoproteasome complex and have downregulated expression of MHC‐I molecules,^[^ ^14^ ^]^ resulting in CTLs losing their target in the tumor microenvironment.^[^ ^5^ ^]^ Therefore, improving the infiltration and tumor recognition ability of CTLs by enhancing the maturation of DCs and upregulating MHC‐I expression in tumor cells will promote the efficacy of PD‐1 inhibitors.

Ginsenoside Rg1 (Rg1) is a tetracyclic triterpenoid in Panax ginseng^[^ ^15^ ^]^ that enhances antigen processing and transport capacity of DCs, and enhance the immune activation effect of DCs. Atractylenolide‐I (Att), a sesquiterpene component in Atractylodes macrocephala, can act on the 26S immunoproteasome complex in MSS CRC cells, thereby upregulating the expression of MHC‐I in the cells.^[^ ^16^ ^]^ Therefore, the combination of Rg1 and Att may promote the infiltration of CTLs into tumor tissues to identify tumor cells by enhancing maturation of DCs and increasing MHC‐I expression in tumor cells. However, owing to the poor bioavailability and differences in physicochemical properties, efficient co‐delivery of the two drugs at the tumor site faces challenges.

Zeolitic imidazolate frameworks (ZIFs) are crystal materials mainly composed of zinc ions and organic ligands connected through coordination bonds, which can improve the bioavailability of drugs.^[^ ^17^ ^]^ It can also release the drug by disassembly in response to the acidic environment of the tumor, achieving controllable drug release in the tumor. ZIF‐90, a classical ZIF configuration,^[^ ^18^ ^]^ is easy to functionalize^[^ ^19^ ^]^ and can achieve efficient co‐loading of multiple drugs.^[^ ^20^ ^]^ Our study demonstrated that aldehyde groups on the surface of ZIF‐90 were oxidized to carboxyl groups to form carboxylated ZIF, which improves the hydrophilicity of the carrier.^[^ ^21^ ^]^ Meanwhile, carboxylated ZIF can co‐load Rg1 and Att with a high drug‐loading capacity and optimize the mass ratio of the two drugs (Rg1/Att‐ZIF).

**Scheme 1:**
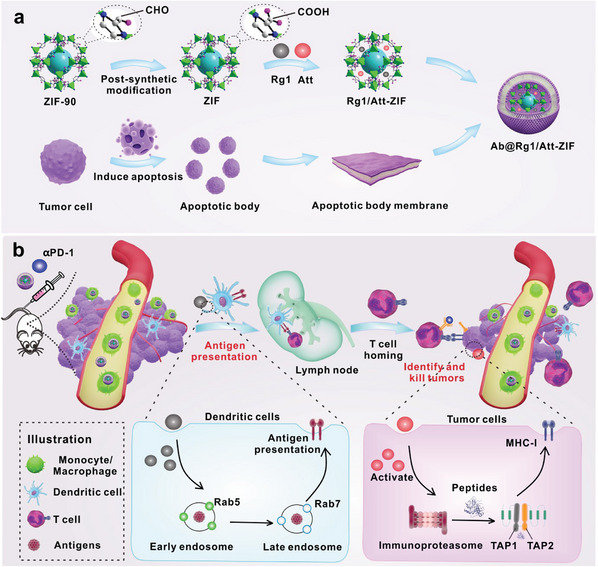
Schematic diagram of Ab@Rg1/Att‐ZIF combined with αPD‐1 in the treatment of MSS CRC. a) Preparation of Ab@Rg1/Att‐ZIF. b) After intravenous injection, Ab@Rg1/Att‐ZIF was phagocytosed by monocytes/macrophages and transported to the tumor center. The disintegration of Ab@Rg1/Att‐ZIF resulted in the release of Rg1 and Att, which promoted the processing and transportation of tumor antigens in DCs, upregulated the expression of MHC‐I in tumor cells, promoted the infiltration of CTLs, and identified and killed tumor cells. Finally, Ab@Rg1/Att‐ZIF was combined with αPD‐1 to treat MSS CRC.

However, as an exogenous nanoparticle, Rg1/Att‐ZIF was easily cleared by the reticuloendothelial system in the circulation in vivo. Barriers such as the dense extracellular matrix, chaotic vascular system, and high interstitial pressure in CRC may further limit the penetration of nanoparticles into the tumor.^[^ ^22^ ^]^ During the development of CRC, circulating monocytes/macrophages in vivo are continuously recruited into the central region of the tumor to differentiate into macrophages, accounting for more than 50% of the number of cells in tumor tissues.^[^ ^23^ ^]^ Therefore, monocytes can be a tool for precise intratumoral drug delivery in CRC.^[^ ^24^ ^]^ Tumor cell apoptotic bodies are 1–5 µm vesicles secreted by tumor cells during apoptosis. They highly express phosphatidylserine (PS) on the membrane surface and can be specifically phagocytosed by inflammatory monocytes.^[^ ^24^ ^]^ Here, we modified tumor cell apoptotic body (Ab) membranes onto the surface of Rg1/Att‐ZIF (Ab@Rg1/Att‐ZIF) for co‐delivery of Rg1 and Att to deep CRC tissues. After intravenous injection, Ab@Rg1/Att‐ZIF was rapidly phagocytosed by inflammatory Ly6C^+^ monocytes, masquerading as a “self” component, evading clearance by the reticuloendothelial system, and efficiently transporting to the tumor center. Ab@Rg1/Att‐ZIF disassembled and released Rg1 and Att under the stimulation of the acidic environment of the tumor. Rg1 and Att significantly regulated maturation in DCs and MHC‐I expression in CRC cells, respectively. Eventually, Ab@Rg1/Att‐ZIF improved the therapeutic efficacy of PD‐1 antibody in MSS CRC by promoting CTL infiltration into the tumor and attacking tumor cells accurately. This study provides novel insights into deep intratumoral delivery and immunotherapy in MSS CRC (**Scheme** **1**).

## 2 Results and Discussion

### 2.1 Maturation of DCs by Rg1 and Upregulation of MHC‐I Expression by Att in CRC Cells In Vitro

**Figure 1:**
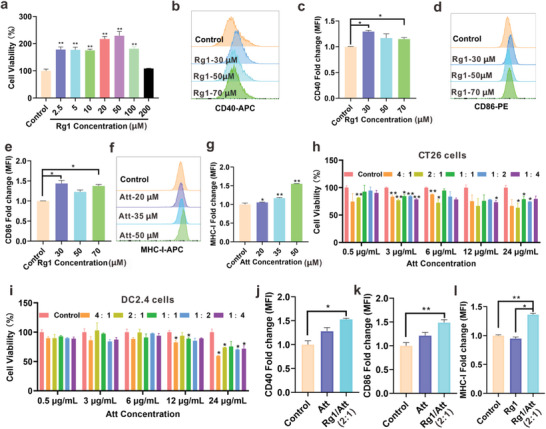
Rg1 improved the maturation of DCs and Att upregulated the expression of MHC‐I in tumor cells. a) Effects of different concentrations of Rg1 on the activity of DC2.4 cells (*n* = 3). b) Flow cytometry and c) quantitative analysis of Rg1 regulating CD40 expression in DC2.4 cells (*n* = 3). d) Flow cytometry and e) quantitative analysis of Rg1 regulating CD86 expression in DC2.4 cells (*n* = 3). f) Flow cytometry and g) quantitative analysis of Att regulating MHC‐I expression in CT‐26 cells. h) Effects of different concentrations and ratios of Rg1 and Att on the activity of CT‐26 cells. i) Effects of different concentrations and ratios of Rg1 and Att on the activity of DC2.4 cells. j) Effects of Rg1 and Att at a mass ratio of 2:1 on the expression of CD40 (k) and CD86 in DC2.4 cells (l) and MHC‐I in CT26 cells (*n* = 3). Data are expressed as the mean ± SD (*p < 0.05; **p < 0.01; ***p < 0.001; one‐way ANOVA).

Infiltration of CTLs into tumor tissue is dependent on the presentation of tumor antigens by mature DCs, whereas DCs function is generally suppressed in the tumor microenvironment.^[^ ^25^ ^]^ In this study, treatment with Rg1 at a concentration of 2.5–100 µm significantly improved proliferation of DC2.4 cells (*p *< 0.01) (**Figure** **1**a). The results of flow cytometry showed that the expression of costimulatory factors (CD40 and CD86) was highest in DC2.4 cells treated with 30 µm Rg1 (Figure 1b–e), indicating that Rg1 promoted DCs maturation. After CTLs are activated and infiltrated into tumors by DCs, they need to bind MHC‐I molecules on tumor cells to recognize and kill tumor cells.^[^ ^26^ ^]^ However, MSS CRC tumor cells are low immunogenic cells, and their surface MHC‐I expression is low.^[^ ^27^ ^]^ We used Att to increase MHC‐I expression in MSS CRC cells and improve their immunogenicity. The expression of MHC‐I was significantly increased in CT‐26 cells when treated with Att (Figure 1f,g), suggesting that tumor cells could be efficiently recognized by CTLs.

Next, we optimized the mass ratio of Rg1 and Att. CCK8 assay was used to verify the effect of the two‐drug combination at different concentrations and ratios on the viability of DC2.4 and CT‐26 cells. The combined use of Rg1 and Att at a mass ratio of 2:1 (Att were 0.5, 3, 6, and 24 µg mL^−1^) significantly inhibited the activity of CT‐26 cells (*p *< 0.01, *p *< 0.05) (Figure 1h) but did not affect the activity of DC2.4 cells (Figure 1i). Meanwhile, the results of flow cytometry showed that the combination of the two drugs at a ratio of 2:1 (Att were 0.5 µg mL^−1^) markedly enhanced the expression of CD40 and CD86 in DC2.4 cells (Figure 1j,k) and promoted the expression of MHC‐I in CT‐26 cells (Figure 1l).

These results demonstrated that the combined use of Rg1 and Att at a mass ratio of 2:1 effectively promoted the maturation of DCs and enhanced MHC‐I expression in CRC cells.

### 2.2 Preparation and Characterization of Ab@Rg1/Att‐ZIF

The increase in hydrophilicity of nanoparticles will prolong their circulation time in vivo.^[^ ^28^ ^]^ To improve the hydrophilicity of the ZIF‐90, we prepared carboxylated ZIF (ZIF) by oxidizing the aldehyde group on the surface of ZIF‐90 into a carboxyl group. The fourier transform infrared (FTIR) spectrum showed a characteristic peak of the carbonyl groups in the enol form at 1600 cm^−1^ (Figure S1, Supporting Information), indicating that carboxyl groups were successfully generated on the surface of ZIF. To encapsulate ZIF with Rg1 and Att at the optimal mass ratio of 2:1, both drugs were loaded at different mass ratios. When Rg1 and Att were loaded at a mass ratio of 10:1, the mass ratio between Rg1 and Att in ZIF was ≈2:1. The drug loading capacity of Rg1 and Att was 15.3 and 8.4 wt.%, respectively (Rg1/Att‐ZIF).

**Figure 2:**
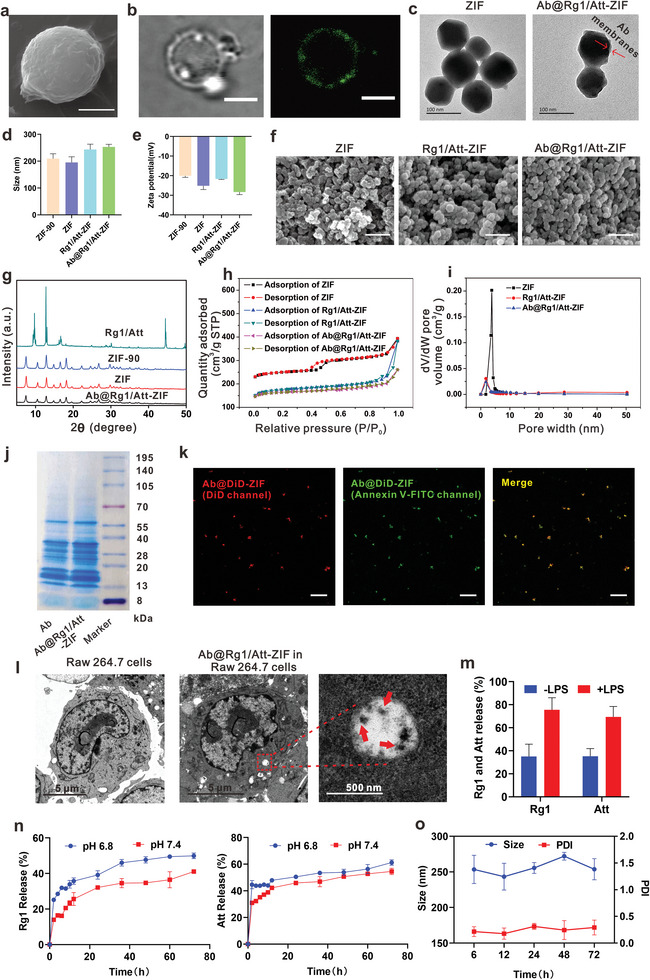
Characterization of Ab@Rg1/Att‐ZIF. a) SEM images of apoptotic bodies. b) CLSM images of apoptotic bodies stained with Annexin V‐FITC (scale bar = 2 µm). c) TEM images of ZIF and Ab@Rg1/Att‐ZIF (scale bar = 100 nm). d) The particle size distribution (e) and zeta potential of ZIF‐90, ZIF, Rg1/Att‐ZIF, and Ab@Rg1/Att‐ZIF were determined via DLS (*n* = 3). f) SEM images of ZIF‐90, ZIF, Rg1/Att‐ZIF, and Ab@Rg1/Att‐ZIF (scale bar = 200 nm). g) XRD patterns of Rg1/Att, ZIF‐90, ZIF, and Ab@Rg1/Att‐ZIF. h) Nitrogen adsorption and desorption isotherms and i) pore size distribution of ZIF, Rg1/Att‐ZIF and Ab@Rg1/Att‐ZIF. j) The protein profiles of Abs and Ab@Rg1/Att‐ZIF were evaluated via SDS‐PAGE. k) CLSM image of Ab@Rg1/Att‐ZIF. ZIF stained with DiD is shown in red, whereas Ab membranes stained with Annexin V‐FITC are shown in green (scale bar = 10 µm). l) Bio‐TEM images of RAW264.7 cells incubated with PBS and Ab@Rg1/Att‐ZIF for 12 h (scale bar: 5 µm and 500 nm, red arrows indicate Ab@Rg1/Att‐ZIF). m) Release of Rg1 and Att from Ab@Rg1/Att‐ZIF‐treated RAW264.7 cells that were cultured in the absence or presence of LPS and IFN‐γ (*n* = 3). n) Effects of physiological and acidic environment on the release of drugs from Ab@Rg1/Att‐ZIF (*n* = 3). o) The stability of Ab@Rg1/Att‐ZIF in PBS solution for 72 h (*n* = 3).

Apoptotic bodies were isolated from apoptotic CT‐26 cells via differential centrifugation. Scanning electron microscopy (SEM) displayed that the apoptotic bodies were spherical vesicles of ≈3 µm (**Figure** **2**a). Confocal laser scanning microscopy (CLSM) showed that the apoptotic bodies had a lipid bilayer structure and phosphatidylserine (PS) was highly expressed on their membrane surface (Figure 2b). PS serves as an “eat me” signal and is specifically phagocytosed by macrophages.^[^ ^29^ ^]^ In addition, the results of flow cytometry exhibited that the proportion of Annexin V‐FITC‐positive apoptotic bodies was 61.9% (Figure S2, Supporting Information). The obtained apoptotic bodies were dispersed in a hypotonic lysis buffer and repeatedly frozen and thawed three times to isolate apoptotic body membranes for subsequent experiments.

Finally, the apoptotic body membranes of CT‐26 cells were coated on the surface of Rg1/Att‐ZIF via ultrasonication (Ab@Rg1/Att‐ZIF). Dynamic light scattering (DLS) revealed that the hydrodynamic size of ZIF, Rg1/Att‐ZIF, and Ab@Rg1/Att‐ZIF were 195.3 ± 21.1, 243.5 ± 19.6, and 253.1 ± 9.8 nm, respectively, and their zeta potential were −25.1 ± 1.9, −21.6 ± 0.2, and −28.3 ± 1.3 mV, respectively (Figure 2d,e). These changes in size and zeta potential confirmed the success of drug encapsulation and Ab membranes modification. SEM and transmission electron microscopy (TEM) showed that Ab@Rg1/Att‐ZIF, ZIF, and ZIF‐90^[^ ^30^ ^]^ were dodecahedrons (Figure 2c,f; Figures S3 and S4, Supporting Information). In addition, the results of SEM and energy spectrum analysis showed that the content of phosphorus was higher in Ab@Rg1/Att‐ZIF than in Rg1/Att‐ZIF, further validating that the coating of Ab membranes was successful (Figure S5, Supporting Information).

Ab@Rg1/Att‐ZIF, ZIF, and ZIF‐90 exhibited the same characteristic peaks in X‐ray diffraction (XRD) spectra, indicating that the three materials had the same crystal structure. The disappearance of the characteristic peaks of Rg1 and Att in Ab@Rg1/Att‐ZIF suggested that the two drugs were encapsulated in ZIF in an amorphous form (Figure 2g). Furthermore, Ab@Rg1/Att‐ZIF, Rg1/Att‐ZIF, ZIF, and ZIF‐90 had type IV nitrogen adsorption and desorption isotherms, with an evident hysteresis loop and a pore size of 1.7 nm (Figure 2h,i; Figure S6, Supporting Information). The BET specific surface areas of ZIF, Rg1/Att‐ZIF and Ab@Rg1/Att‐ZIF were 765.04, 551.65 and 505.42 m^2^ g^−1^, respectively. This decrease in BET specific surface area was attributed to the encapsulation of Rg1 and Att in pores and the coating of Ab membranes on the surface.

Sodium dodecyl sulfate‐polyacrylamide gel electrophoresis (SDS‐PAGE) revealed that the overall protein composition of Abs and Ab@Rg1/Att‐ZIF was similar (Figure 2j). CLSM showed that DiD‐labeled ZIF (red) and FITC‐Annexin‐labeled Ab membranes (green) were colocalized. This result indicated that Ab membranes were successfully coated on Rg1/Att‐ZIF, and PS was highly expressed on the surface of Ab@Rg1/Att‐ZIF (Figure 2k). Bio‐TEM showed that the nanoparticle morphology could still be observed at 12 h after Ab@Rg1/Att‐ZIF was internalized by RAW264.7 (Figure 2l). After Ab@Rg1/Att‐ZIF were internalized by RAW264.7 cells for 12 h, 75.5% of Rg1 and 69.4% of Att were released into the lipopolysaccharide (LPS) and interferon (IFN)‐γ induced inflammatory environment (Figure 2m), suggesting that macrophages that internalize nanoparticles can release the drug when stimulated by the tumor inflammatory environment.^[^ ^31^ ^]^ To determine the form of drug released by RAW264.7 cells, we used Att as an index component to determine the percentage of free drug in the inflammatory environment. The percentage of free drug release was 69.3%, whereas the remaining 30.7% of the drug was released in the form of nanoparticles. In vitro experiments demonstrated that Ab@Rg1/Att‐ZIF disintegrated and released Rg1 and Att in the acidic environment of tumor tissues (pH = 6.8)^[^ ^32^ ^]^ (Figure 2n). These results suggest that macrophages may release Rg1 and Att in the form of free drugs or nanoparticles, and the released nanoparticles respond to the acidic tumor environment to further release the drug.

We determined the biological stability of Ab@Rg1/Att‐ZIF, the samples were stored in 1 × phosphate‐buffered saline (PBS) (pH 7.4) and fetal bovine serum (FBS) and their size and PDI were measured over time. The particle size and polymer dispersity index (PDI) of Ab@Rg1/Att‐ZIF did not change significantly until 72 h (Figure 2o; Figure S7, Supporting Information), indicating that Ab@Rg1/Att‐ZIF remained stable for 3 days under normal physiological conditions.

### 2.3 Regulation of Endosomal Transport in DCs and Immunoproteasome in Tumor Cells by Ab@Rg1/Att‐ZIF In Vitro

**Figure 3:**
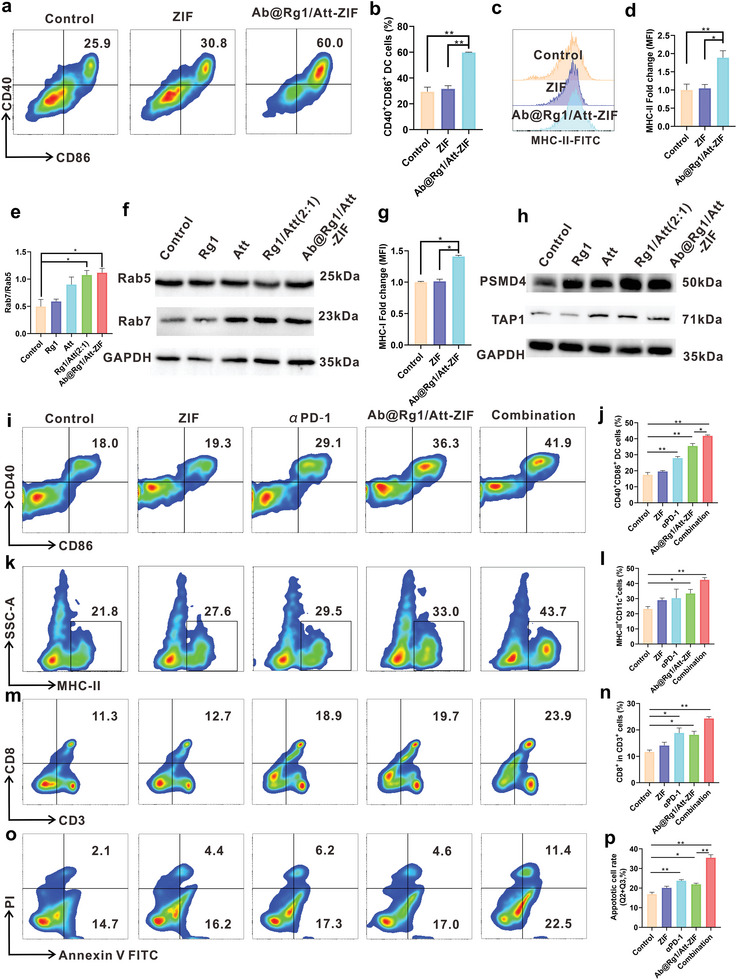
Ab@Rg1/Att‐ZIF regulated antigen presentation ability of DC cells and upregulated MHC‐I expression in tumor cells. a) Flow cytometry and b) quantitative analysis of Ab@Rg1/Att‐ZIF regulating CD40 and CD86 expression in BMDCs in the co‐culture system (*n* = 3). c) Flow cytometry and d) quantitative analysis of Ab@Rg1/Att‐ZIF regulating MHC‐II expression in BMDCs in the co‐culture system (*n* = 3). e) Quantitative analysis and f) western blotting was performed to evaluate the expression of Rab7/Rab5 (*n* = 3). g) Effects of Ab@Rg1/Att‐ZIF on the expression of MHC‐I in CT‐26 cells. h) Western blotting was performed to evaluate the expression of PSMD4 and TAP1. i) Flow cytometry and j) quantitative analysis of CD11c^+^MHC‐II^+^ DCs in splenic immune cells co‐incubated with CT‐26 cells (*n* = 3). k) Flow cytometry and l) quantitative analysis of CD11c^+^CD86^+^ DCs in splenic immune cells co‐incubated with CT‐26 cells (*n* = 3). m) Flow cytometry and n) quantitative analysis of CD3^+^CD8^+^ T cells in splenic immune cells co‐incubated with CT‐26 cells (*n* = 3). o) Flow cytometry and p) quantitative analysis of apoptosis levels of CT‐26 cells in splenic immune cells co‐incubated with CT‐26 cells (*n* = 3). All data are expressed as the mean ± SD (*p < 0.05; **p < 0.01; ***p < 0.001; one‐way ANOVA).apoptosis levels of 4T1 cells.

To assess whether Ab@Rg1/Att‐ZIF could promote the maturation of DCs, a co‐culture model of DC2.4/bone marrow‐derived dendritic cells (BMDCs) and CT‐26 cells was established using transwell chambers. The results of flow cytometry demonstrated that Ab@Rg1/Att‐ZIF upregulated the expression of CD40 and CD86 in DCs (**Figure** **3**a,b; Figure S8, Supporting Information) and promoted the maturation of DCs. Meanwhile, we also found that Ab@Rg1/Att‐ZIF significantly enhanced MHC‐II expression (antigen‐presenting molecule) in BMDCs (Figure 3c,d). Furthermore, the mechanisms of Ab@Rg1/Att‐ZIF enhancing the maturation of DCs were examined. The antigen‐presenting ability of DCs is closely related to their endosomal antigen transport.^[^ ^33^ ^]^ In the tumor microenvironment, tumor antigens engulfed by DCs are processed and presented to T cells as Rab5‐expressing early endosomes transform into Rab7‐expressing late endosomes mature.^[^ ^10^ ^]^ In this study, Western blotting and CLSM revealed that Ab@Rg1/Att‐ZIF group significantly increased the Rab7/Rab5 ratio of DCs (*p *< 0.05) (Figure 3e,f; Figure S9, Supporting Information), which was consistent with the results reported in the literature.^[^ ^10^ ^]^ Altogether, the results showed that Ab@Rg1/Att‐ZIF increased the ratio of Rab7/Rab5 in DCs, enhanced antigen processing and transport ability of DCs, and promoted DCs maturation.

Besides the level of infiltration of CTLs, infiltrating CTLs also need to bind antigens presented by MHC‐I in tumor cells to effectively recognize and kill tumor cells.^[^ ^34^ ^]^ The results of flow cytometry showed that Ab@Rg1/Att‐ZIF significantly increased the expression of MHC‐I in CT‐26 cells and HLA‐I in HT‐29 cells (human‐derived MSS CRC cells) (*p *< 0.05) (Figure 3g; Figure S10, Supporting Information), which facilitated the killing of tumor cells by CTLs. We further studied the mechanism of Ab@Rg1/Att‐ZIF up‐regulating expression of MHC‐I in tumor cells. MHC‐I expression in tumor cells requires processing of MHC‐I‐associated antigenic peptides by the proteasome 26S subunit non‐ATPase 4 (PSMD4) and transporting antigenic peptides to MHC‐I by downstream transporter associated with antigen processing (TAP) proteins, such as TAP1, which ultimately promoting MHC‐I up‐regulation.^[^ ^35^ ^]^ Western blotting revealed that Ab@Rg1/Att‐ZIF enhanced the expression of PSMD4 and TAP1 in CT‐26 cells (Figure 3h), indicating that Ab@Rg1/Att‐ZIF promoted the expression of MHC‐I by promoting the generation of more antigenic peptides from the tumor cell immunoproteasome and transport them through TAP1.

To validate our hypothesis that Ab@Rg1/Att‐ZIF enhances T cell recognition and killing of tumor cells to improve the efficacy of PD‐1 antibodies, we constructed a co‐culture system consisting of CT‐26 tumor cells and splenic immune cells. Flow cytometry analysis showed that Ab@Rg1/Att‐ZIF significantly increased the expression of CD40, CD86, and MHC‐II in DCs. The combination of Ab@Rg1/Att‐ZIF and PD‐1 antibodies further enhanced the expression of CD40, CD86, and MHC‐II in DCs (Figure 3i–l). Due to the increasing proportion of mature DCs in the co‐cultivation system, we have also observed an increase in the proportion of CD8^+^CD3^+^ T cells in the combination group (Figure 3m,n). At the same time, there is a significant increase in the proportion of apoptotic tumor cells in the combination group (Figure 3o,p).

In summary, Ab@Rg1/Att‐ZIF can promote DCs maturation and increase MHC‐I expression in tumor cells, thereby facilitating CTL recognition and killing of tumor cells to improve the therapeutic efficacy of PD‐1 antibodies.

### 2.4 Transport of Ab@Rg1/Att‐ZIF to DCs and Tumor Cells by Ly‐6C^+^ Monocytes

**Figure 4:**
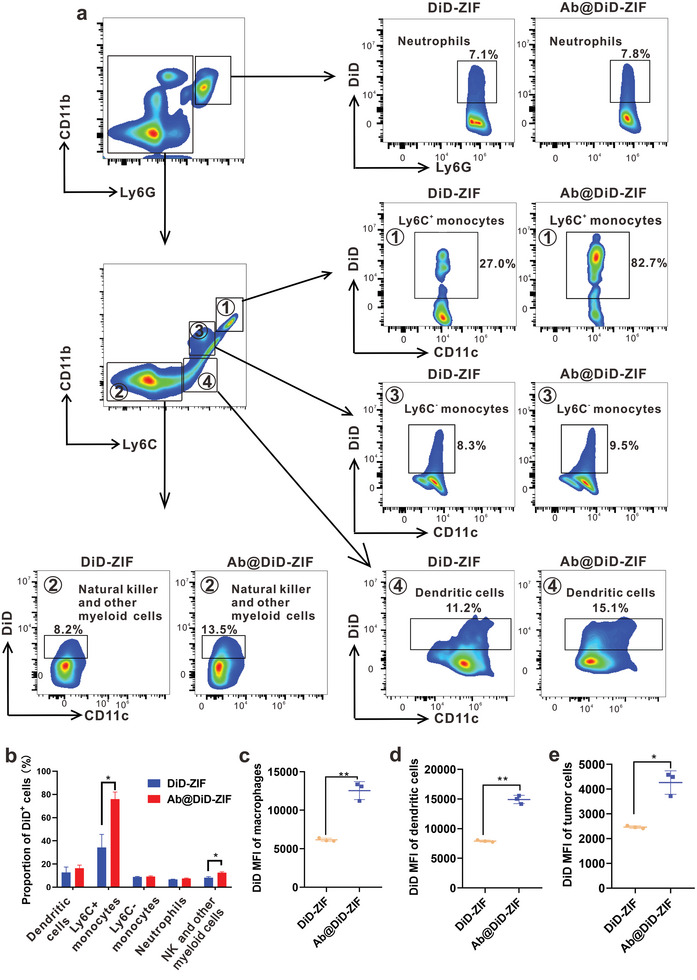
Internalization of Ab@Rg1/Att‐ZIF by monocytes/macrophages in vivo and delivery of drugs to DCs and tumor cells. a) Flow cytometry was performed to assess the selective uptake of DiD‐ZIF or Ab@DiD‐ZIF by PBMCs. b) Proportion of DiD in PBMCs. c) Fluorescence intensity of DiD in macrophages. d) Fluorescence intensity of DiD in DCs. e) Fluorescence intensity of DiD in tumor cells. All data are expressed as the mean ± SD (*p < 0.05; **p < 0.01; ***p < 0.001; two‐tailed Student's t‐test).

Ly‐6C^+^ monocytes are classical inflammatory monocytes that specifically phagocytose apoptotic bodies by recognizing PS on their surface.^[^ ^24^ ^]^ Meanwhile, they are continuously recruited to tumor tissues during the development of CRC.^[^ ^36^ ^]^ To assess whether Ab@Rg1/Att‐ZIF can be specifically engulfed by Ly‐6C^+^ monocytes in the blood, fluorescence‐activated cell sorting (FACS)^[^ ^37^ ^]^ was used to identify mouse peripheral blood mononuclear cells (PBMCs) subsets that engulfed Ab@DiD‐ZIF or DiD‐ZIF (**Figure** **4**a).

As shown in Figure 4b, ≈76% of Ab@DiD‐ZIF and only 34% of DiD‐ZIF were internalized by inflammatory Ly‐6C^+^ monocytes (CD11b^+^ Ly‐6C^+^), indicating that Ab‐coated nanoparticles were specifically phagocytosed by Ly‐6C^+^ monocytes. Besides, compared with DiD‐ZIF, Ab@DiD‐ZIF was significantly internalized by natural killer cells and other myeloid cells (≈12%) (*p *< 0.05). These cell subsets have the characteristics of chemotaxis to tumor tissues and good tumor‐penetrating ability^[^ ^38^ ^]^ and serve as an effective tool for intratumoral administration of Ab@Rg1/Att‐ZIF. The internalization of DiD‐ZIF and Ab@DiD‐ZIF by DCs, neutrophils, and Ly‐6C^−^ monocytes were low, and no significant differences were observed in the uptake rate of the two materials among the three cell subsets (Figure 4b).

When nanoparticles are internalized by Ly‐6C^+^ monocytes in circulation, they will be carried to tumor tissues with the tumor chemotaxis of Ly‐6C^+^ monocytes.^[^ ^24^ ^]^ Upon stimulation by the tumor inflammatory environment, Ly‐6C^+^ monocytes release nanoparticles and are taken up by other cells in the microenvironment.^[^ ^31^, ^39^ ^]^ We determined the fluorescence intensity of DiD in monocytes/macrophages, DCs, and tumor cells to assess whether Ab@Rg1/Att‐ZIF is taken up by DCs and tumor cells after being released by Ly‐6C^+^ monocytes in tumor tissues. Compared with the DiD‐ZIF group, the Ab@DiD‐ZIF group exhibited a 2.07‐, 1.88‐, and 1.73‐fold increase in the fluorescence intensity of DiD in macrophages, DCs, and tumor cells, respectively (Figure 4c–e; Figure S11, Supporting Information). These results validated that Ab@DiD‐ZIF was specifically internalized by circulating inflammatory monocytes/macrophages into tumors and delivered to intratumoral DCs and tumor cells.

### 2.5 Tumor Targeting and Intratumoral Penetration

**Figure 5:**
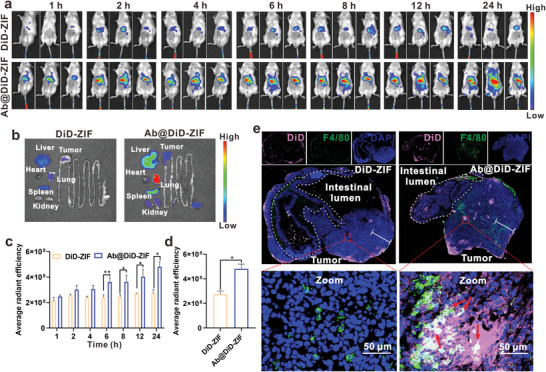
Biodistribution of Ab@Rg1/Att‐ZIF. a) The distribution of DiD‐ZIF and Ab@DiD‐ZIF in mice with CRC was visualized using the IVIS Spectrum. b) After 24 h, the ex vivo imaging of various organs and tumor tissues was performed using the IVIS Spectrum. c) Fluorescence intensity of tumor tissues at different time points (*n* = 3). d) Quantitative analysis of fluorescence intensity in isolated tumor tissues (*n* = 3). e) CLSM was used to observe the penetration of Ab@DiD‐ZIF into tumor tissues and their co‐localization with macrophages (scale bar = 2 mm). All data are expressed as the mean ± SD. (*p < 0.05; **p < 0.01; ***p < 0.001; two‐tailed Student's t‐test).

During the development of CRC, inflammatory monocytes will be continuously recruited into the tumor microenvironment, which will lead to the accumulation of nanoparticles in tumor tissues.^[^ ^40^ ^]^ We used near‐infrared in vivo imaging to verify whether Ab@Rg1/Att‐ZIF can continuously accumulate in tumor tissues. The results revealed that the fluorescence intensity of Ab@DiD‐ZIF in the tumor site gradually increased, and the fluorescence intensity at 6, 8, 12, and 24 h was significantly stronger than that of DiD‐ZIF (*p *< 0.05, *p *< 0.01) (**Figure** **5**a,c). After 24 h, tumor tissues and major organs were harvested from mice for fluorescence quantification. The fluorescence intensity at the tumor site in the Ab@DiD‐ZIF group was 1.77 times higher than that in the DiD‐ZIF group (Figure 5b,d; Figure S12, Supporting Information). These results confirmed that Ab@DiD‐ZIF had better tumor‐targeting ability owing to their efficient internalization by monocytes/macrophages.

The dense extracellular matrix, chaotic vascular system, and high interstitial pressure formed during the development of CRC greatly hinder the penetration of nanoparticles into the tumor.^[^ ^2^ ^]^ Biologically active monocytes/macrophages can penetrate into the center of the tumor tissue to differentiate into macrophages.^[^ ^24^ ^]^ We analyzed the frozen sections of mouse tumor tissues to assess the intratumoral penetration ability of Ab@Rg1/Att‐ZIF. The results showed that the pink fluorescence signal in the DiD‐ZIF group was localized only in the superficial part of the tumor, and that in the Ab@DiD‐ZIF group was observed to have penetrated the central region of the tumor (Figure 5e). In addition, the pink fluorescence signal in the Ab@DiD‐ZIF group was strongly colocalized with the green fluorescence of macrophages (Figure 5e). These results suggested that Ab@DiD‐ZIF were transported to the internal region of the tumor via inflammatory monocytes/macrophages.

### 2.6 Synergistic Efficacy of Ab@Rg1/Att‐ZIF and αPD‐1 in an Orthotopic MSS CRC Transplantation Mouse Model

**Figure 6:**
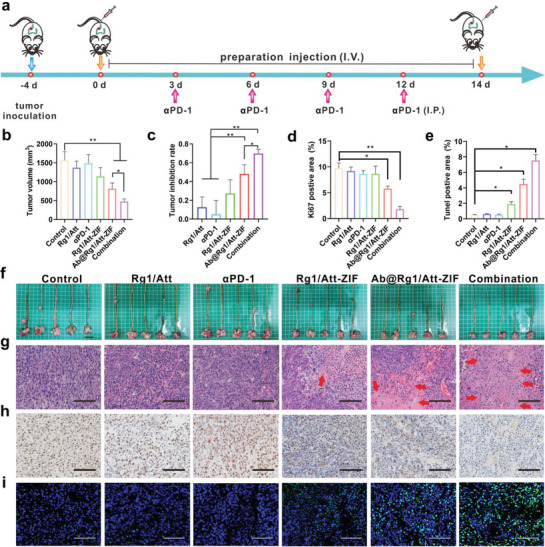
Ab@Rg1/Att‐ZIF combined with αPD‐1 in the treatment of MSS CRC. a) The treatment regimen of the combination of Ab@Rg1/Att‐ZIF and αPD‐1 in MSS CRC. b) Tumor volume (*n* = 5), c) tumor inhibition rate (*n* = 5) after treatment in each group. d) Quantitative analysis of Ki67 and e) Tunel positive area after treatment in each group (*n* = 3). f) The images of tumors after treatment in each group (*n* = 5, scale bar = 1 cm). g) H&E staining, h) Ki67 staining, i) and Tunel staining of tumor tissues after treatment in each group (Red arrows indicate tumor cell necrosis, nuclear fragmentation or dissolution disappeared, scale bar = 100 µm). All data are expressed as the mean ± SD (*p < 0.05; **p < 0.01; ***p < 0.001; one‐way ANOVA).

An orthotopic mouse model of MSS CRC was established to examine the therapeutic effects of Ab@Rg1/Att‐ZIF combined with αPD‐1. The specific administration regimen is shown in **Figure** **6**a. The combination of Ab@Rg1/Att‐ZIF and αPD‐1 markedly inhibited tumor growth (*p *< 0.01), whereas αPD‐1 had a weak ability to inhibit tumor growth (Figure 6b,f). The tumor inhibition rate was ≈69% in the combination group and only 5% in the αPD‐1 group (Figure 6c). The observed poor efficacy of αPD‐1 in this study is consistent with clinical reports that in a phase II clinical trial on pembrolizumab (a PD‐1 inhibitor), patients with MSS CRC were not found to respond to pembrolizumab.^[^ ^41^ ^]^

Hematoxylin and eosin (H&E) staining revealed that the tumor necrosis area was high in the combination group, whereas the tumor necrosis area was small and anti‐tumor effects were weak in the αPD‐1 group (Figure 6g). Ki67 and TUNEL staining showed that tumor proliferation was significantly inhibited and the degree of tumor cell apoptosis was increased in the combination group (Figure 6d,e,h,i). Altogether, the combined use of Ab@Rg1/Att‐ZIF and αPD‐1 greatly improved the efficacy of αPD‐1 in the treatment of MSS CRC.

### 2.7 Ab@Rg1/Att‐ZIF Promoted CTL Infiltration by Enhancing the Maturation of DCs In Vivo

**Figure 7:**
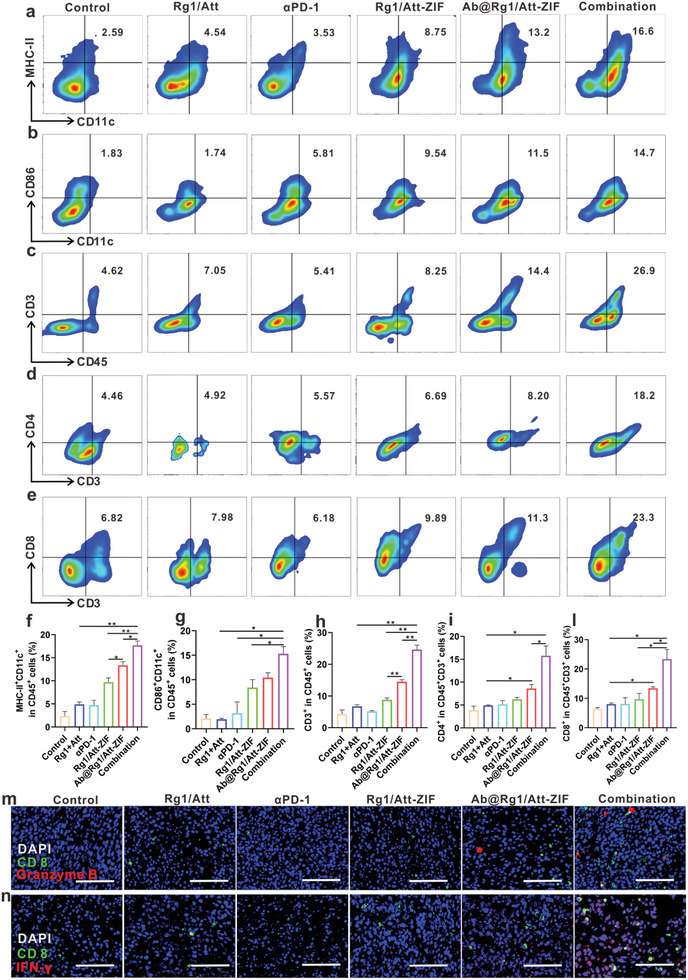
Ab@Rg1/Att‐ZIF regulated DCs to increase the infiltration levels of CTLs. a) Flow cytometry was used to evaluate the number of CD11c^+^CD86^+^ DCs, b) CD11c^+^MHC‐II^+^ DCs, c) CD45^+^CD3^+^ T cells, d) CD3^+^CD4^+^ T cells, and e) CD3^+^CD8^+^ T cells. f) Quantification of CD11c^+^MHC‐II^+^ DCs, g) CD11c^+^CD86^+^ DCs, h) CD45^+^CD3^+^ T cells, i) CD3^+^CD4^+^ T cells, and l) CD3^+^CD8^+^ T cells (*n* = 3). m) Number of granzyme B^+^CD8^+^ T cells and n) IFN‐γ^+^CD8^+^ T cells (scale bar = 100 µm). All data are expressed as the mean ± SD (*p < 0.05; **p < 0.01; ***p < 0.001; one‐way ANOVA).

The anti‐tumor effects of αPD‐1 rely on the number of infiltrated CTLs. However, CTLs infiltration requires mature DCs to present phagocytosed tumor antigens to naive T cells.^[^ ^42^ ^]^ We analyzed the immune status of DCs in tumor tissues via flow cytometry to assess whether Ab@Rg1/Att‐ZIF could improve the immune stimulation ability of DCs. As shown in **Figure** **7**a,f and Figure S13 (Supporting Information), the proportion of CD45^+^CD11c^+^CD86^+^ DCs was 5.2‐ and 7.6‐fold higher in the Ab@Rg1/Att‐ZIF and combination groups than that in the control group, respectively. Similarly, compared with the control group, the proportion of CD45^+^CD11c^+^MHC‐II^+^ DCs exhibited 5.7‐ and 7.6‐fold higher in the Ab@Rg1/Att‐ZIF and combination groups, respectively (Figure 7b,g; Figure S13, Supporting Information). These results suggest that Ab@Rg1/Att‐ZIF promotes the maturation of DCs in tumor tissues. Importantly, the combination group had a higher proportion of mature DCs, which would further facilitate T‐cell infiltration. At the same time, we determined the proportion of tumor‐infiltrating T lymphocytes in the combined treatment group. Consistently, a significant increase in CD45^+^CD3^+^ T cells in the combination group compared with those in the control group (Figure 7c,h; Figure S13, Supporting Information). Among them, CD45^+^CD3^+^CD4^+^ T cells increased 4.1‐fold and CD45^+^CD3^+^CD8^+^ cells increased 3.7‐fold in the combined group compared to the control group (Figure 7d,e,i,l; Figure S13, Supporting Information). To further analyze whether recruited tumor‐infiltrating T lymphocytes had tumor‐killing capacity, we measured the proportion of T cells expressing granzyme B (killer tumor protein) in tumor tissue. Immunofluorescence analysis revealed that more CTLs with tumor‐killing ability (granzyme B^+^ CD8 T cells and IFN‐γ^+^ CD8 T cells) were observed in the combination group (Figure 7m,n).

Collectively, Ab@Rg1/Att‐ZIF improved the therapeutic efficacy of αPD‐1 in MSS CRC by enhancing the antigen‐presenting ability of DCs and recruiting more CTLs to infiltrate tumor tissues.

### 2.8 Ab@Rg1/Att‐ZIF Upregulated MHC‐I Expression in Tumor Cells by Acting on Immunoproteasomes In Vivo

**Figure 8:**
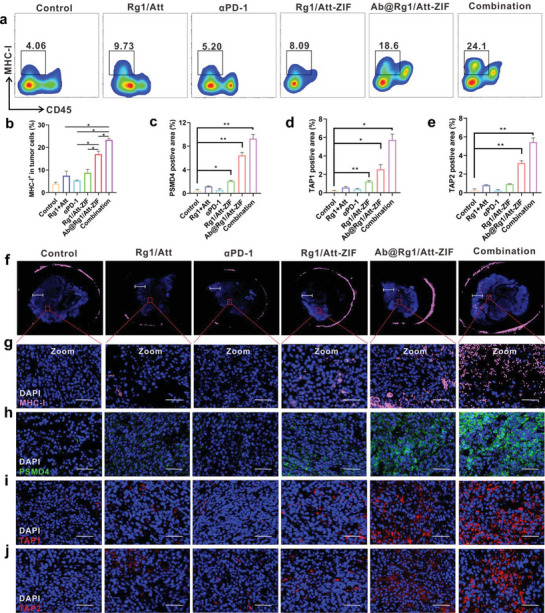
Ab@Rg1/Att‐ZIF upregulated the expression of MHC‐I in tumor cells. a,b) Flow cytometry was used to evaluate the number of MHC‐I^+^ tumor cells. c) Quantitative analysis of PSMD4, d) TAP1 and e) TAP2 postive area after treatment in each group (*n* = 3). f) CLSM was used to evaluate the number of MHC‐I^+^ tumor cells in tumor tissues of each group after treatment (scale bar = 2 mm). g) CLSM was used to assess the local amplification of MHC‐I^+^ tumor cells in tumor tissues (scale bar = 50 µm). h) CLSM was used to assess the expression of PSMD4, i) TAP1, and j) TAP2 in tumor tissues (scale bar = 50 µm). All data are expressed as the mean ± SD (*p < 0.05; **p < 0.01; ***p < 0.001; one‐way ANOVA).

The infiltration of CTLs into tumor tissues requires their binding to MHC‐I on the surface of tumor cells to effectively recognize the cells and kill the cells.^[^ ^43^ ^]^ We used the flow cytometry to determine the expression of MHC‐I in tumor tissues. As shown in **Figure** **8**a,b, the proportion of MHC‐I^+^ tumor cells was 4.4‐ and 6.1‐fold higher in the Ab@Rg1/Att‐ZIF and combination groups than that in the control group, respectively. This increased proportion of MHC‐I^+^ tumor cells helps infiltrating CTLs to effectively identify and kill tumor cells. In addition, immunofluorescence analysis revealed that the pink fluorescence signal of MHC‐I was stronger in the central region of tumor tissues in the Ab@Rg1/Att‐ZIF and combination groups (Figure 8f,g). These results confirmed that the delivery strategy mediated by monocytes/macrophages successfully transported drugs to the deep tumor tissues.

Furthermore, the mechanism of Ab@Rg1/Att‐ZIF promoting MHC‐I expression in tumor tissues was examined. PSMD4 in tumor cells mediates the recruitment of ubiquitinated proteins for degradation and antigen processing,^[^ ^44^ ^]^ downstream TAP1 and TAP2 transport the processed antigenic peptides to MHC‐I to promote its expression.^[^ ^35^ ^]^ Immunofluorescence analysis revealed that the expression of PSMD4, TAP1, and TAP2 was significantly high in tumor cells in the combination group (Figure 8c–e,h–j). These results indicated that Ab@Rg1/Att‐ZIF up‐regulates MHC‐I expression in tumor cells by acting on tumor immunoproteasomes and facilitates CTLs to recognize and kill tumors, thereby improving the efficacy of αPD‐1 for MSS CRC.

### 2.9 Safety of the Combination Therapy of Ab@Rg1/Att‐ZIF and αPD‐1

**Figure 9:**
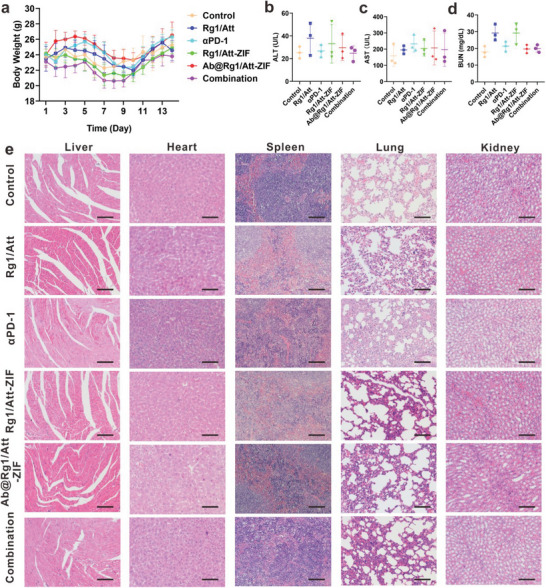
In vivo safety. a) Changes in the body weight of mice in each group during treatment and the serum levels of (b) ALT, c) AST, d) and BUN after treatment (*n* = 3). e) H&E staining of major mouse organs after treatment in each group (scale bar = 100 µm). All data are expressed as the mean ± SD (*p < 0.05; **p < 0.01; ***p < 0.001; one‐way ANOVA).

First, we found that Ab@Rg1/Att‐ZIF did not undergo hemolysis by hemolysis experiments (Figure S14, Supporting Information). To evaluate the biocompatibility of Ab@Rg1/Att‐ZIF and its combination with αPD‐1, the body weight of mice was monitored daily during the treatment period. The results showed that there were no significant changes in body weight among the six groups (**Figure** **9**a), indicating that long‐term administration of Ab@Rg1/Att‐ZIF and combination treatment did not have harmful effects on mice. Consistently, no significant differences in the serum levels of ALT, AST, and BUN were observed among the six groups (Figure 9b–d), demonstrating that Ab@Rg1/Att‐ZIF and combination treatment did not affect liver or kidney function. In addition, H&E staining showed no lesions in the major organs of mice (Figure 9e). Altogether, the combination of Ab@Rg1/Att‐ZIF and αPD‐1 was safe.

## 3 Conclusion

This study presents a nanoplatform for monocyte/macrophage‐mediated transport in vivo to enhance the therapeutic effects of αPD‐1 against MSS CRC. The use of in vivo monocytes/macrophages as drug delivery tools for Ab@Rg1/Att‐ZIF overcame the drug delivery barrier of the CRC microenvironment, thus realizing efficient accumulation and deep penetration of drugs in tumor tissues. Ab@Rg1/Att‐ZIF promoted the infiltration of CTLs into tumor tissues to precision attack tumor cells by promoting the maturation of DCs and enhancing the immunoproteasome activity in tumor cells. Eventually, the combined use of Ab@Rg1/Att‐ZIF and αPD‐1 remarkably improved the efficacy of αPD‐1 in the treatment of MSS CRC.

## 4 Experimental Section

### Materials

Zinc acetate [ZnCH_3_(COO)_2_·2H_2_O, purity 99%] and hydrogen peroxide (H_2_O_2_, ≈30 wt.% in H_2_O) were obtained from Aladdin (Shanghai, China). Imidazole‐2‐carboxaldehyde (2‐ICA, purity 99%), trioctylamine, and DiD were purchased from Shanghai Yien Chemical Technology Co., Ltd. (Shanghai, China). Rg1, Att, and staurosporine (purity >98%) were purchased from Shanghai Yuanye Biotechnology Co., Ltd. (Shanghai, China). Lipopolysaccharide (LPS) and 4′, 6‐diamidino‐2‐phenylindole (DAPI) were obtained from Biosharp Biotechnology (Hefei, China). Ficoll‐Paque PREMIUM 1.084 sterile solution was purchased from Cytiva (USA).

### Antibodies

Anti‐mouse PD‐1 mAb was purchased from BioXcell Co., Ltd. (New Hampshire, USA). Fixable Viability Dye eFluor 506 (65 086 614) and anti‐CD45‐FITC (2 312 013), anti‐CD16/32 (2 439 142), anti‐CD3‐PE (2 313 178), anti‐CD4‐PE‐Cy7 (2 363 818), anti‐CD8a‐APC (2 356 251), anti‐CD45‐FITC (2 312 013), anti‐H2k^d^‐eFlour450 (2 082 380), anti‐CD45‐eFlour450 (242 398), anti‐CD11c‐PE‐Cy5.5 (2 383 313), anti‐CD11b‐eFlour780 (2 481 282), anti‐IA/IE‐Alexa Flour700 (2 410 914), anti‐CD206‐APC (2 506 988), and anti‐Ly6C‐PE (2 442 222) antibodies were purchased from eBioscience (California, USA). Anti‐CD86‐PE (B357977), anti‐CD40‐APC (B351629), and anti‐H^2^kd‐APC (B323588) antibodies were obtained from BioLegend (California, USA). Anti‐Ly6G‐Elab Flour Violet 450 antibody was obtained from Elabscience (Wuhan, China).

### Cell Lines

The mouse MSS CRC cell line CT‐26 was obtained from the National Collection of Authenticated Cell Culture (Shanghai, China). CT‐26 cells were cultured in RPMI 1640 medium supplemented with 10% fetal bovine serum (FBS) and 1% penicillin/streptomycin at 37 °C in a humidified atmosphere with 5% CO_2_. The mouse immature DC line DC2.4 was purchased from Shanghai Fuheng Biotechnology Co., Ltd. (Shanghai, China). DC2.4 cells were cultured in RPMI 1640 medium supplemented with 10% FBS at 37 °C in a humidified atmosphere with 5% CO_2_. RAW264.7 cells were retained in the laboratory and cultured in DMEM supplemented with 10% FBS and 1% penicillin/streptomycin under standard conditions.

### Animals

Six‐week‐old male Balb/c mice (weight, 20 ± 2 g) were obtained from Nanjing Jicu Yaokang Technology Co., Ltd. All animal handling procedures were approved by the Animal Ethics Committee of Jiangsu Academy of Traditional Chinese Medicine (AEWC‐20220920‐235).

### Cell Viability

DC2.4 cells were seeded in 96‐well plates (1 × 10^4^ cells well^−1^) and cultured for 24 h. Subsequently, the culture medium was removed, and the cells were exposed to Rg1 at various concentrations (2.5, 5, 10, 20, 50, 100, and 200 µm). After 24 h of incubation, cell viability was assessed via CCK8 assay.

To investigate the effects of different concentrations and ratios of Rg1 and Att on the viability of DC2.4 and CT‐26 cells, both cell lines were seeded in 96‐well plates at a density of 1 × 10^4^ cells well^−1^ and treated with Rg1 and Att solutions at mass ratios of 4:1, 2:1, 1:1, 1:2, and 1:4 (Att were 3, 6, 12, and 24 µg mL^−1^). After 24 h of incubation, cell viability was assessed via CCK8 assay.

### Preparation of Carboxylated ZIF

Zn(CH_3_COO)_2_·2H_2_O (0.176 g, 2 mL) and 2‐ICA (0.154 g, 8 mL) in DMF solution were mixed slowly and stirred for 5 min. Trioctylamine (600 µL) was added to the solution, followed by continuous stirring for 12 h. Finally, the product was collected via centrifugation (12,000 rpm) for 30 min, washed with excess ethanol, and vacuum‐dried at 40 °C to obtain ZIF‐90. To prepare carboxylated ZIF, ZIF‐90 was dispersed in 15 mL of water with 0.12 mmoL hydrogen peroxide and stirred for 24 h. Thereafter, the mixture was freeze‐dried to obtain carboxylated ZIF (ZIF). The surface carboxyl groups of ZIF were characterized via Fourier transform infrared (FT‐IR) spectroscopy (Nicolet 6700, Thermo Fisher Scientific, USA).

### Extraction and Characterization of Tumor Apoptotic Bodies and Their Membranes

CT‐26 cells were seeded in culture dishes and incubated with 10 µm staurosporine for 3 h to induce apoptosis. Tumor apoptotic bodies were extracted via differential centrifugation as follows: centrifugation at 200 g for 10 min, collection of the supernatant, centrifugation at 2000 g for 20 min, and collection of precipitates as apoptotic bodies. The morphological features of the apoptotic bodies were observed using scanning electron microscopy (SEM; ZEISS EVO 18, UK). PS located on the surface of the apoptotic bodies was labeled with FITC‐Annexin and observed using confocal laser scanning microscopy (CLSM; Leica TCS SP8, Germany). In addition, the yield of apoptotic bodies labeled with FITC‐Annexin was quantitatively determined via flow cytometry.

The apoptotic bodies were dispersed in a hypotonic lysis buffer composed of 10 mm tris (pH 7.4), 10 mm magnesium chloride, and 1 mm benzylsulfonyl fluoride. Subsequently, they were repeatedly frozen and thawed thrice, centrifuged at 10,000 g for 10 min, and washed thrice with ultrapure water to obtain apoptotic body (Ab) membranes.

### Preparation of Ab@Rg1/Att‐ZIF

Rg1 (18 mg) was added to 3 mL of a methanol solution containing ZIF (3 mg) and stirred for 12 h in the dark. Att (1.8 mg) was added to the solution, followed by continuous stirring for 12 h in the dark. Subsequently, the solution was centrifuged to obtain ZIF co‐loaded with Rg1 and Att (Rg1/Att‐ZIF). The drug‐loading capacity of Rg1/Att‐ZIF was determined via high‐performance liquid chromatography (HPLC; Wates 2695, USA). Eventually, Ab membranes and Rg1/Att‐ZIF were added to PBS at a mass ratio of 1:1 and sonicated for 5 min in an ice bath to obtain Rg1/Att‐ZIF coated with Ab membranes (Ab@Rg1/Att‐ZIF).

### Characterization of Ab@Rg1/Att‐ZIF

The morphological features of Ab@Rg1/Att‐ZIF were observed using SEM and transmission electron microscopy (TEM). The specific surface area and pore structure of Ab@Rg1/Att‐ZIF were assessed using an automatic specific surface area and porosity analyzer. The crystal structure of Ab@Rg1/Att‐ZIF was examined using an X‐ray diffractometer (D8 Advance, Bruker, Karlsruhe, Germany). The scanning range was 5–50°, and the scanning speed was 5° min^−1^. After the fusion of FITC‐Annexin‐labeled Ab membranes and DiD‐labeled ZIF, the expression of PS on the surface of Ab@Rg1/Att‐ZIF was analyzed via CLSM. A rapid one‐step whole‐protein staining method was used to map proteins in the samples. The stability of the Ab@Rg1/Att‐ZIF in PBS and FBS was monitored via dynamic light scattering (DLS) for 72 h at room temperature.

### Drug Release In Vitro

RAW264.7 cells (3 × 10^5^ cells well^−1^) were incubated with Ab@Rg1/Att‐ZIF (200 µg mL^−1^) for 2 h, and the medium was replaced with a fresh medium containing LPS (1 µg mL^−1^) and IFN‐γ (200 ng mL^−1^). After 12 h of incubation, the content of drugs in the culture medium was measured to assess drug release. The medium was centrifuged at 5500 rpm for 15 min, and the content of free drug in the supernatant was evaluated.

To assess drug release in the tumor acid environments, Ab@Rg1/Att‐ZIF was placed in PBS solutions with pH 6.8. The solutions were collected at various time points and replaced with fresh solutions. The collected solutions were analyzed on a Waters QDA Detector. The mobile phase involved a mixture of 0.1% phosphate buffer and acetonitrile (0–3 min, 75:25 v/v; 3–18 min, 75:25–10:90 v/v; 18–20 min, 10:90–75:25 v/v; 20–24 min, 75:25 v/v), pumped at a flow rate of 1 mL min^−1^ through the column (Supersil ODS2, 5 µm, 150 × 4.6 mm).

### Maturation of DCs In Vitro

DC2.4 cells were seeded in 12‐well plates (1 × 10^5^ cells well^−1^) and cultured for 24 h. The medium was replaced with 30‐, 50‐, or 70‐µm Rg1 prepared in the tumor‐conditioned medium. After 24 h of incubation, the cells were harvested, washed with PBS, incubated with anti‐CD86‐PE and anti‐CD40‐APC antibodies for 0.5 h, and analyzed via flow cytometry.

To establish a co‐culture system of DCs and tumor cells, DC2.4 cells/BMDCs were inoculated in the lower transwell chamber and CT‐26 cells were inoculated in the upper transwell chamber. After 24 h of culture, the cells were incubated with PBS, Att, Rg1/Att (2:1), ZIF, or Ab@Rg1/Att‐ZIF for 24 h. Subsequently, the expression of CD40 and CD86 on DC2.4 cells was measured via flow cytometry.

To assess endosomal maturation, DC2.4 cells were incubated with PBS, Att, Rg1/Att (2:1), or Ab@Rg1/Att‐ZIF for 24 h, and the expression of Rab5a and Rab7 was analyzed using CLSM and Western blotting.

### Expression of MHC‐I in Tumor Cells In Vitro

CT‐26 cells were seeded in 12‐well plates (5 × 10^4^ cells well^−1^) and cultured for 24 h. After the culture medium was removed, the cells were incubated with Att at various concentrations (20, 35, and 50 µm) for 24 h. Subsequently, the cells were harvested, washed with PBS, incubated with anti‐H^2^kd‐APC antibodies for 0.5 h, and analyzed via flow cytometry.

Furthermore, CT‐26/HT‐29 cells were incubated with PBS, Rg1, Rg1/Att (2:1), ZIF, or Ab@Rg1/Att‐ZIF for 24 h, and the expression of MHC‐I/HLA‐I was measured via flow cytometry.

### Western Blotting

CT‐26 cells were incubated with PBS, Rg1, Att, Rg1/Att (2:1), or Ab@Rg1/Att‐ZIF for 24 h. The cells were collected, and total proteins were extracted using RIPA buffer (Beyotime). Subsequently, the expression of PSMD4 and TAP1 was analyzed via western blotting.

### Analysis of DCs‐Mediated T Cell Killing of Tumor Cells

CT‐26 cells (1 × 10^5^ cells well^−1^) and immune cells extracted from the spleen of mouse (1 × 10^6^ cells well^−1^) were co‐inoculated in the 6‐well plate overnight. Subsequently, the medium was replaced with fresh culture medium containing PBS, ZIF, αPD‐1, Ab@Rg1/Att‐ZIF, and Ab@Rg1/Att‐ZIF+αPD‐1. After incubation for 24 h, the floating immune cells were stained with anti‐CD11c‐PE‐Cy5, anti‐CD86‐PE, and anti‐CD40‐APC, anti‐CD3‐FITC, and anti‐CD8‐APC antibodies and analyzed via flow cytometry. Apoptosis kit was used to analyze the proportion of tumor cell apoptosis.

### Monocyte/Macrophage Transport Preparation In Vivo

To establish an in situ model of MSS CRC, CT‐26 cells (1 × 10^6^ cells) were injected into the right armpit of male Balb/c mice. When the diameter of the solid tumor reached 1 cm after 14 d, the mice were sacrificed and tumor tissues were harvested and cut into 1‐mm^3^ blocks. The mice were anesthetized with isoflurane, and the intestines were scratched gently. The tumor block was fixed to the intestinal wound using a matrix gel. After the matrix gel dried, the external intestinal segment was included in the abdomen and sutured surgically.

Ab@ZIF or ZIF loaded with DiD (10 µg) were injected into the tail vein of mice. After 2 h of injection, whole blood was collected from mice in the presence of an anticoagulant, and peripheral blood mononuclear cells (PBMCs) were extracted using Ficoll‐PaqueTM PREMIUM. After the non‐specific binding was blocked using FC antibody, the purified PBMCs were stained with anti‐CD11c‐PE‐Cy7, anti‐CD11b‐PE‐Cy5.5, anti‐Ly6G‐Elab Flour Violet 450, and anti‐Ly6C‐PE antibodies and analyzed via flow cytometry.

### Distribution and Intercellular Transport of Ab@Rg1/Att‐ZIF

DiD‐labeled ZIF or Ab@ZIF were injected into the tail vein of mice with MSS CRC. IVIS was used to observe the fluorescence intensity in vivo at different time points (1, 2, 4, 6, 8, 12, and 24 h). After 24 h, the mice were sacrificed and the fluorescence intensity of the heart, liver, spleen, lung, kidney, and tumor was observed using IVIS. To verify the intratumoral penetration ability of Ab@ZIF, tumor tissues were frozen‐sectioned, stained with F4/80 antibody and DAPI, and analyzed via CLSM.

Tumor tissues were prepared into a single‐cell suspension, and non‐specific binding was blocked using FC antibody. Subsequently, the cells were incubated with anti‐CD45‐FITC, anti‐CD11c‐PE‐Cy7, and anti‐CD11b‐PE‐Cy5.5 antibodies and analyzed via flow cytometry.

### Anti‐Tumor Assessment In Vivo

To evaluate the anti‐tumor effects of Ab@Rg1/Att‐ZIF combined with αPD‐1, tumor‐bearing mice were randomly divided into 6 groups as follows: PBS, αPD‐1, Rg1/Att, Rg1/Att‐ZIF, Ab@Rg1/Att‐ZIF, and Ab@Rg1/Att‐ZIF + αPD‐1. αPD‐1 (0.1 mg) was administered intraperitoneally every 3 days, the remaining treatments were administered once daily via tail vein injection (Rg1, 2.25 mg kg^−1^; Att, 1.25 mg kg^−1^). The body weight of mice was recorded daily. After 14 days of treatment, the mice were sacrificed and serum was separated. In addition, tumors and major organs were collected, weighed, and photographed. Tumor volume was calculated as follows: width^2^ × length × 0.5. The tumor tissues were fixed in 4% formalin; embedded in paraffin; and sliced for H&E, Ki67, and Tunel staining.

### Analysis of Tumor‐Infiltrating T Cells and DCs

After the mice were sacrificed, tumor tissues were collected and prepared into single‐cell suspensions. Non‐specific binding was blocked using FC antibodies, and the cells were stained with Fixable Viability Dye eFluor 506 and anti‐CD45‐FITC, anti‐CD3‐PE, anti‐CD4‐PE‐Cy7, anti‐CD8a‐APC, and anti‐H2k^d^‐eFlour450 antibodies to evaluate the proportion of CTLs and the expression of MHC‐I in tumor cells. In addition, the cells were stained with Fixable Viability Dye eFluor 506 and anti‐CD45‐eFlour450, anti‐CD11c‐PE‐Cy5.5, anti‐IA/IE‐Alexa Flour700, anti‐CD206‐APC, and anti‐CD86‐PE antibodies to assess DCs maturation.

### Immune Mechanism Analysis

Mouse tumor tissue sections were stained with MHC‐I, CD8, granzyme B, IFN‐γ, PSMD4, TAP1, and TAP2 antibodies to evaluate the anti‐tumor mechanisms of Ab@Rg1/Att‐ZIF.

### In Vivo Safety Assessment

To evaluate the safety of Ab@Rg1/Att‐ZIF in vivo, the major organs of mice were subjected to H&E staining. The serum levels of AST, ALT, and BUN were measured to evaluate the potentially toxic effects of Ab@Rg1/Att‐ZIF on the liver and kidney.

### Statistical Analysis

All statistical analyses were performed using the GraphPad Prism 8.0 software (GraphPad Software, San Diego, CA, USA). The two‐tailed Student's t‐test and one‐way ANOVA were used to analyze significant differences. A P‐value of <0.05 was considered statistically significant.

## Conflict of Interest

The authors declare no conflict of interest.

## Supporting information

Supporting Information

## Data Availability

The data that support the findings of this study are available from the corresponding author upon reasonable request.
