# Supplementary material for: Monocyte/Macrophage‐Mediated Transport of Dual‐Drug ZIF Nanoplatforms Synergized with Programmed Cell Death Protein‐1 Inhibitor Against Microsatellite‐Stable Colorectal Cancer
Source: Adv Sci (Weinh). 2024 Aug 5;11(38):2405886. doi: 10.1002/advs.202405886 (PMC11481235; doi:10.1002/advs.202405886)
Supplement: Supplementary file 1 — Supporting Information [file ADVS-11-2405886-s001.docx]

**Monocyte/Macrophage-Mediated Transport of Dual-Drug ZIF Nanoplatforms Synergized with Programmed Cell Death Protein-1 Inhibitor Against Microsatellite-Stable Colorectal Cancer**

*Xietao Ye, Yuping Liu, Liangyin Wei, Yeyang Sun, Xiaoran Zhang, Hong Wang, Hong Guo, Xiaoying Qin, Xiaoqi Li, Ding Qu*, Jiege Huo*, and Yan Chen**

X Ye and Y Liu contributed equally.

X Ye, Y Liu, L Wei, Y Sun, X Zhang, H Wang, H Guo, X Qin, X Li, D Qu, J Huo, Y Chen

Affiliated Hospital of Integrated Traditional Chinese and Western Medicine, Nanjing University of Chinese Medicine, Nanjing 210028, China

E-mail: quding@jsatcm.com, huojiege@jsatcm.com, [chenyan@jsatcm.com](mailto:ychen202@hotmail.com)

X Ye, Y Liu, L Wei, Y Sun, X Zhang, H Wang, H Guo, X Qin, D Qu, X Li, Y Chen

Multi-component of Traditional Chinese Medicine and Microecology Research Center, Jiangsu Province Academy of Traditional Chinese Medicine, Nanjing 210028, China

Y Liu, J Huo, Y Chen

Jiangsu Clinical Innovation Center of Digestive Cancer of Traditional Chinese Medicine, Nanjing 210028, China


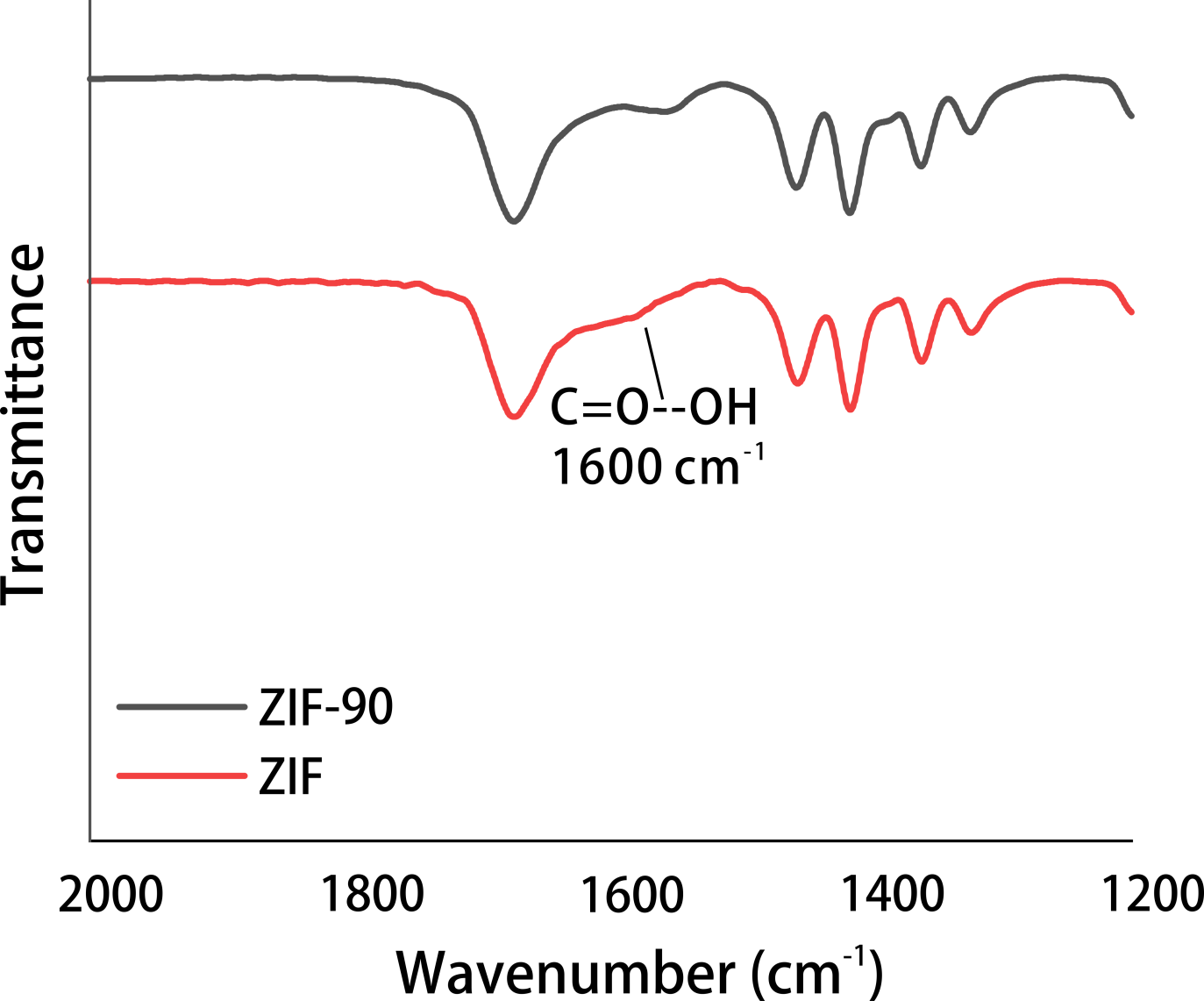


Figure S1. Fourier Transform Infrared Spectra of ZIF-90 and ZIF.


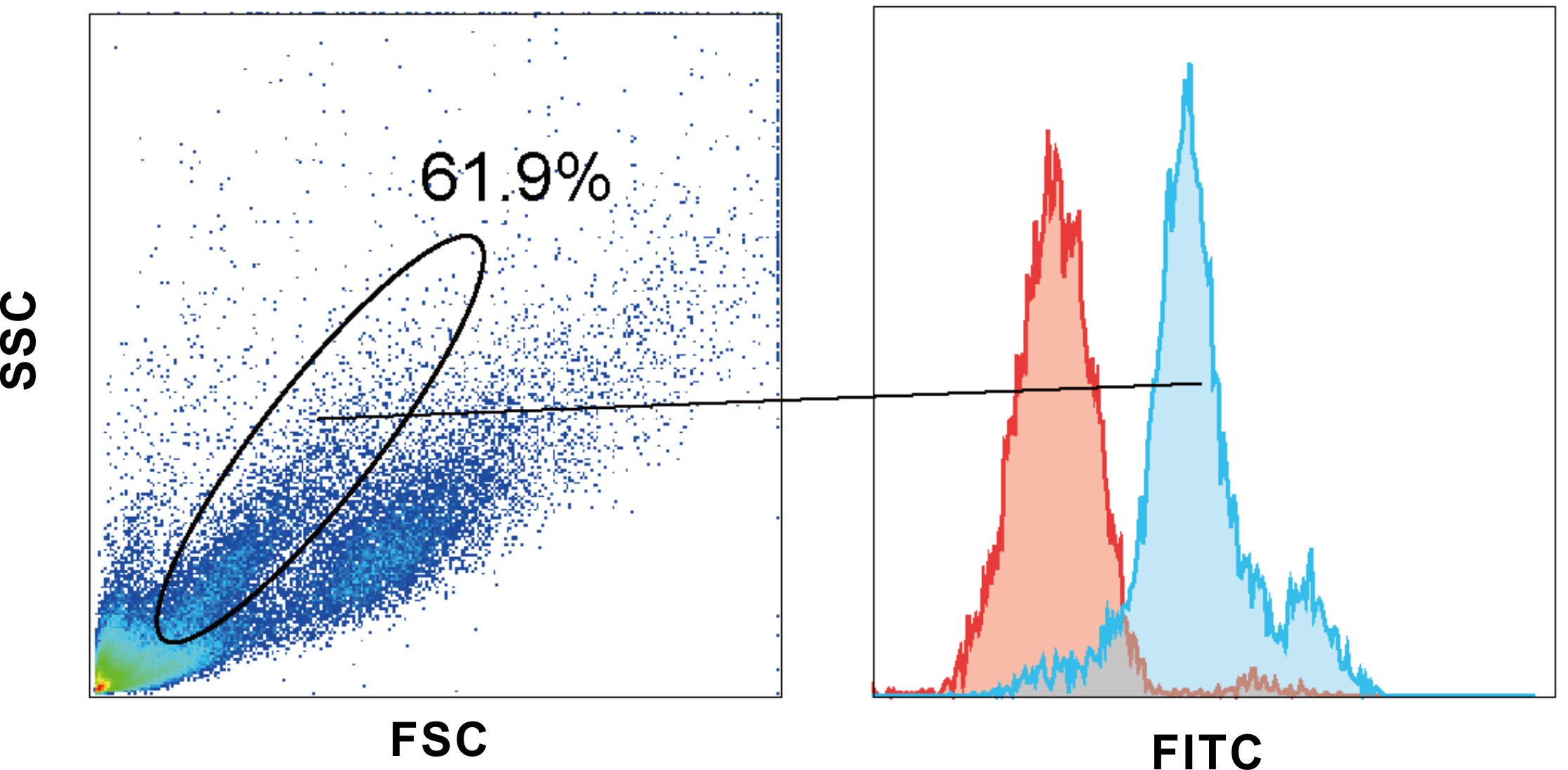


Figure S2. Flow cytometry images of apoptotic body stained with Annexin V-FITC


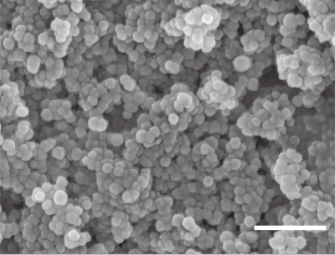


Figure S3. SEM images of ZIF-90 (scale bar = 200 nm).


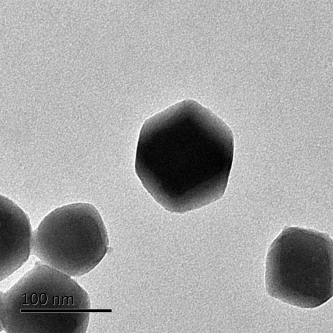


Figure S4. TEM images of ZIF-90 (scale bar = 100 nm).


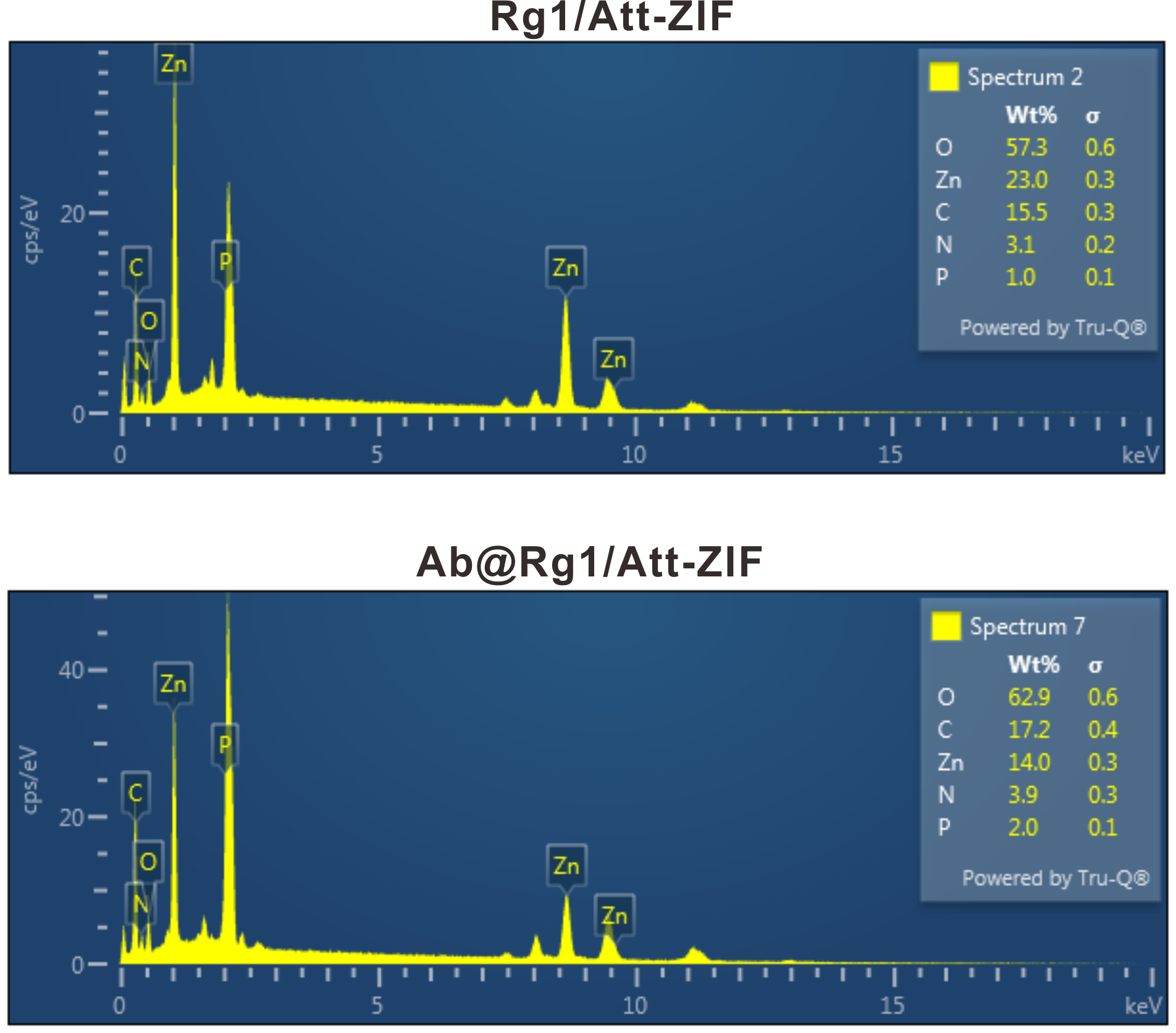


Figure S5. Elemental composition analysis of Rg1/Att-ZIF and Ab@Rg1/Att-ZIF was conducted using SEM coupled with energy-dispersive X-ray spectroscopy (SEM-EDS).


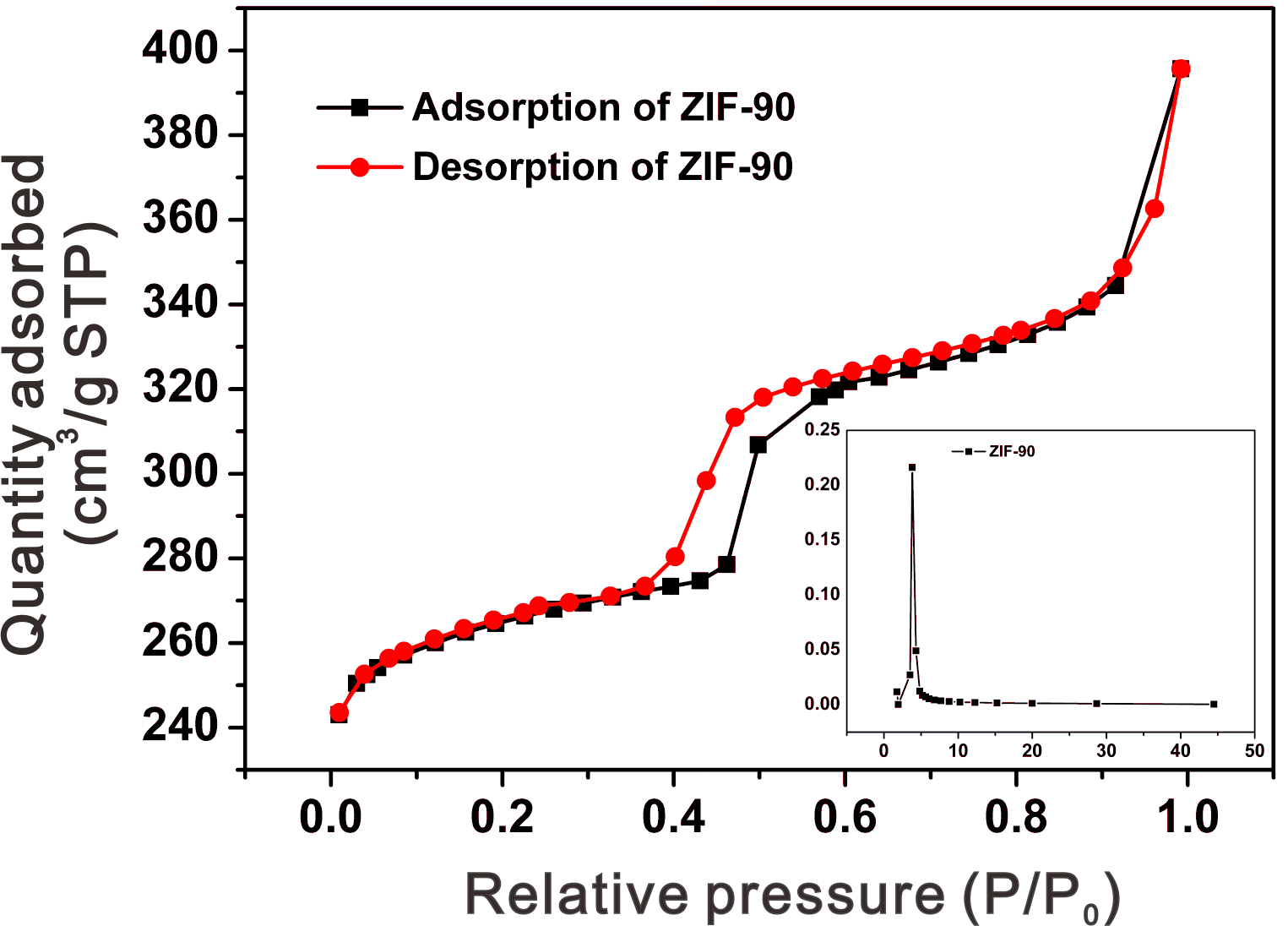


Figure S6. Nitrogen adsorptionedesorption isotherm and pore size distribution of ZIF-90.


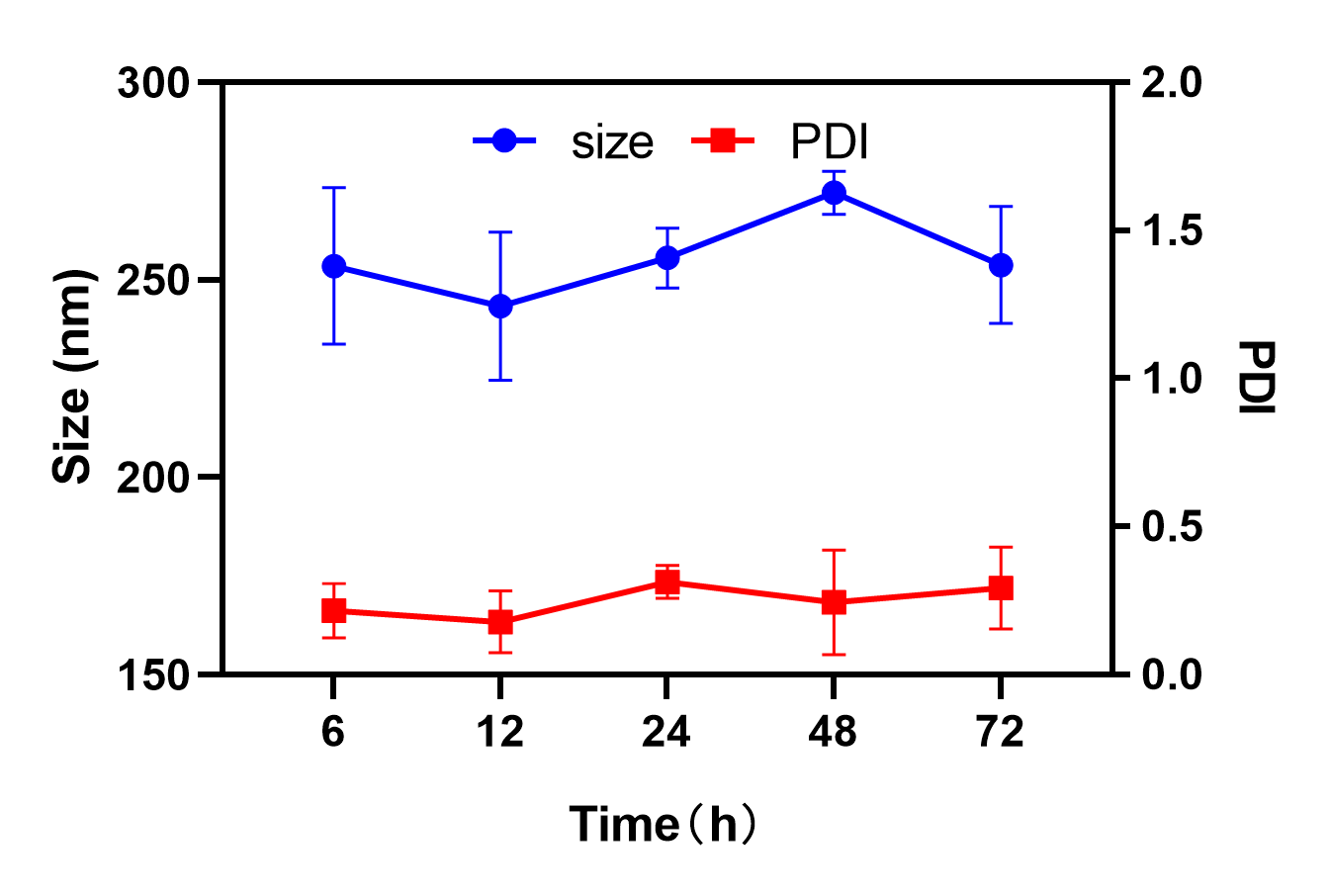


Figure S7. The stability of Ab@Rg1/Att-ZIF in FBS solution for 72 h (n = 3).


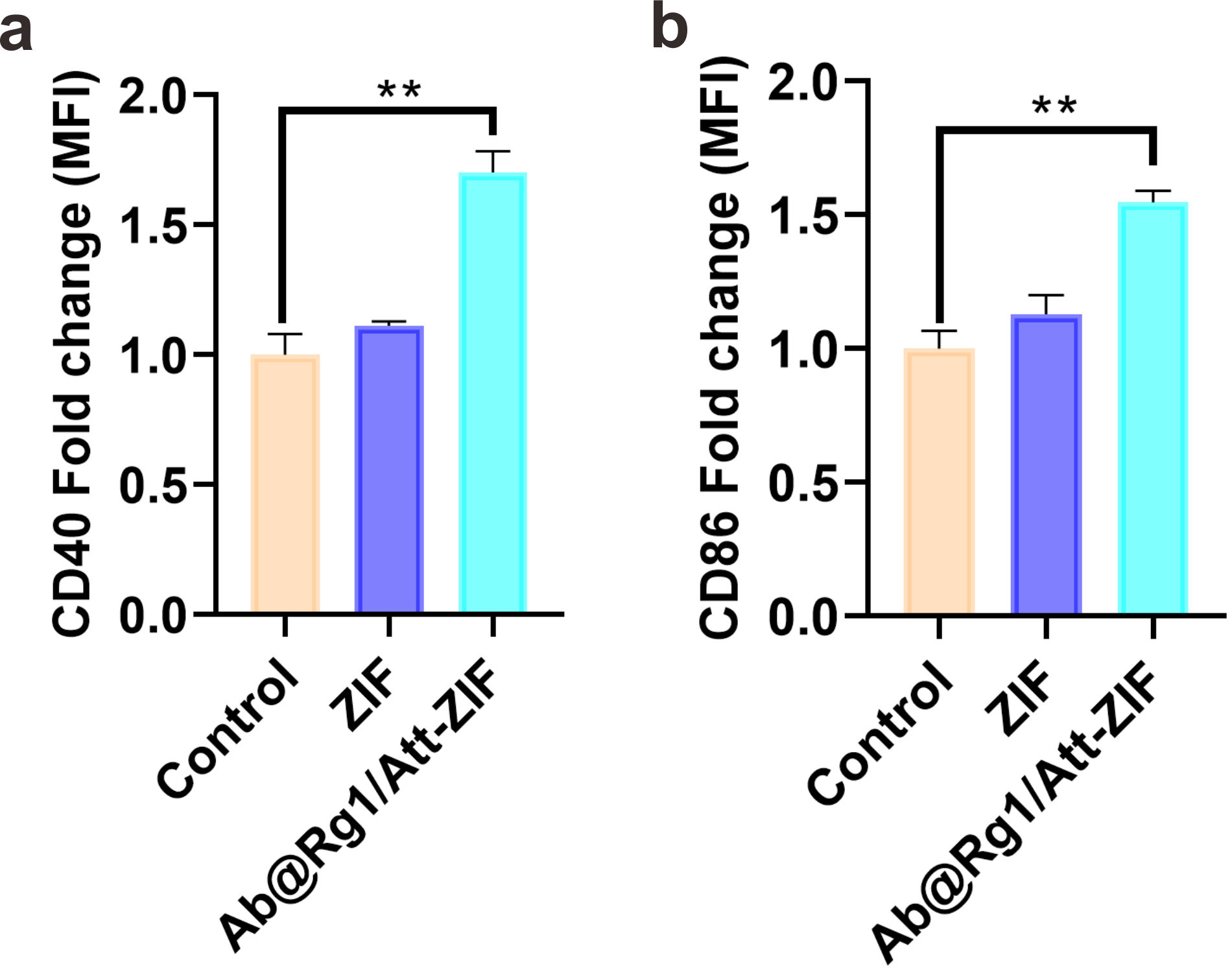


Figure S8. (a) Flow cytometry and quantitative analysis of Ab@Rg1/Att-ZIF regulating CD40 and (b) CD86 expression in DC2.4 cells in the co-culture system (n = 3).


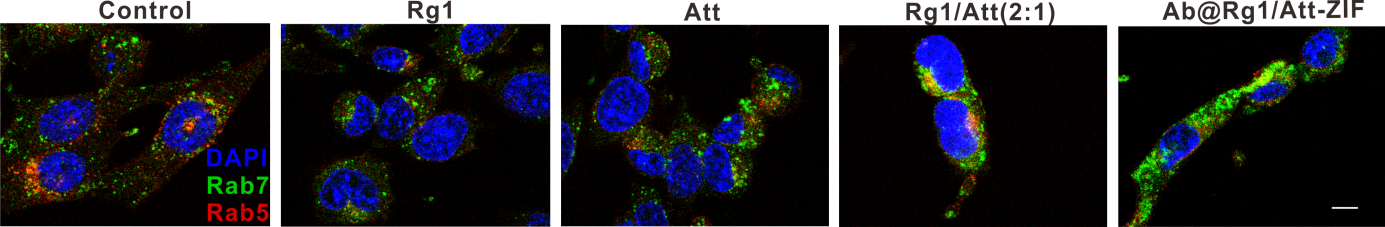


Figure S9. (a) CLSM was performed to evaluate the expression of Rab7/Rab5 (n = 3, scale bar = 10 μm).


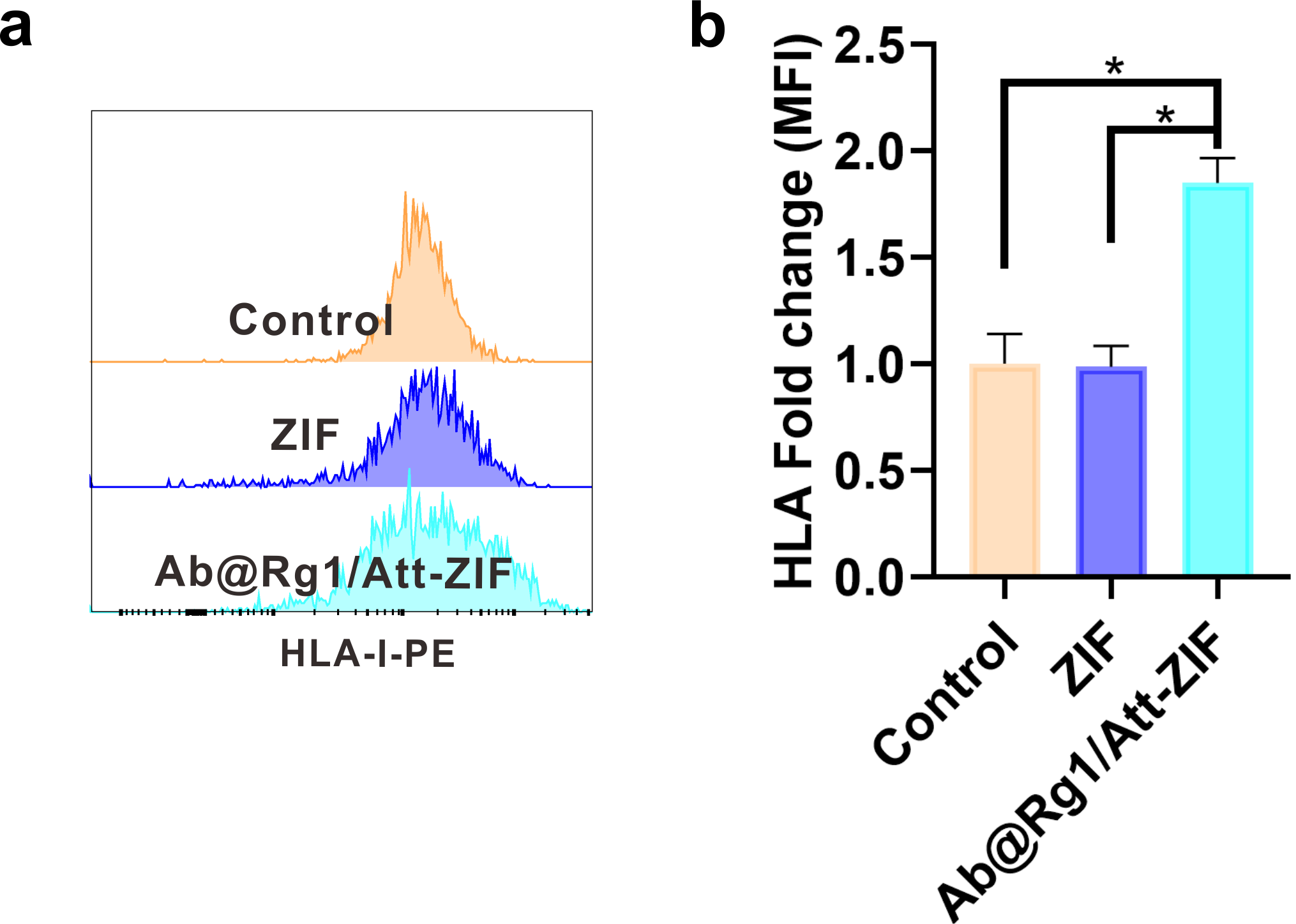


Figure S10. (a) Flow cytometry and quantitative analysis of Ab@Rg1/Att-ZIF regulating HLA-I expression in HT29 cells (n = 3).


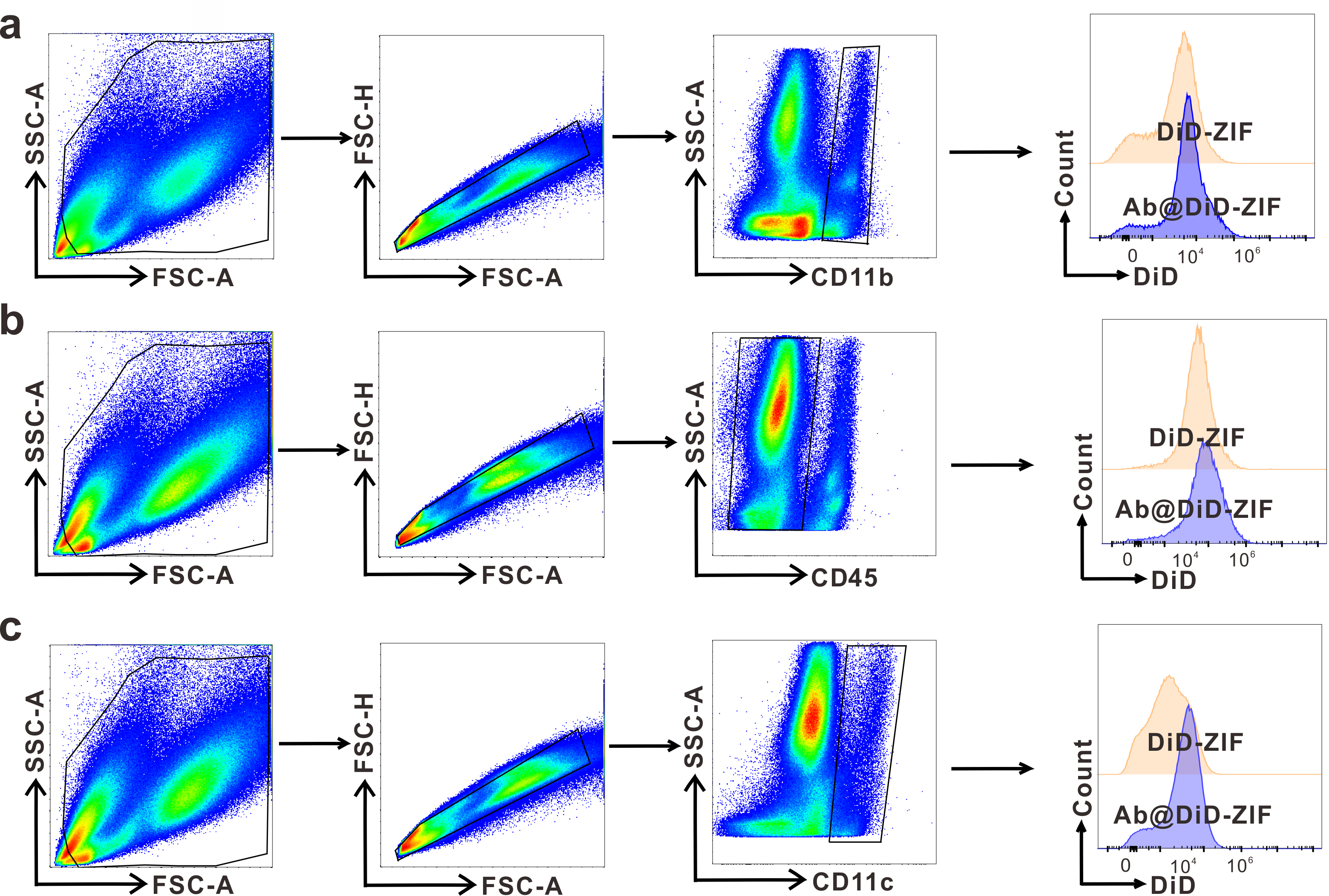


Figure S11. (a) The fluorescence intensity of DiD in macrophages. (b) The fluorescence intensity of DiD in DCs. (c) The fluorescence intensity of DiD in tumor cells (n=3).


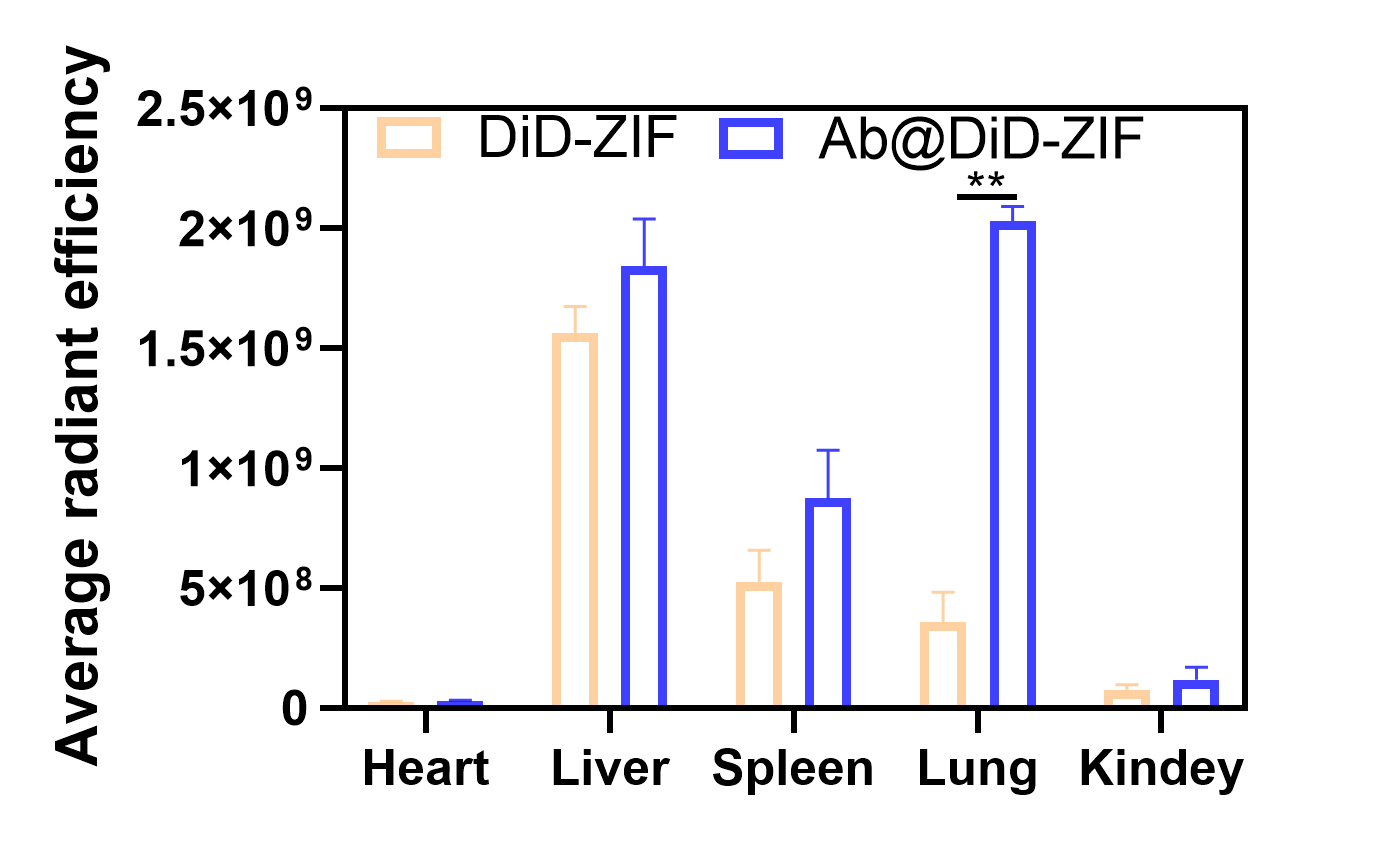


Figure S12. Quantitative results of fluorescence intensity in isolated heart, liver, spleen, lung and kidney tissues (n=3). Data represent the means ± SD. **p* < 0.05, ***p* < 0.01, ****p* < 0.001, two-tailed student t-test.


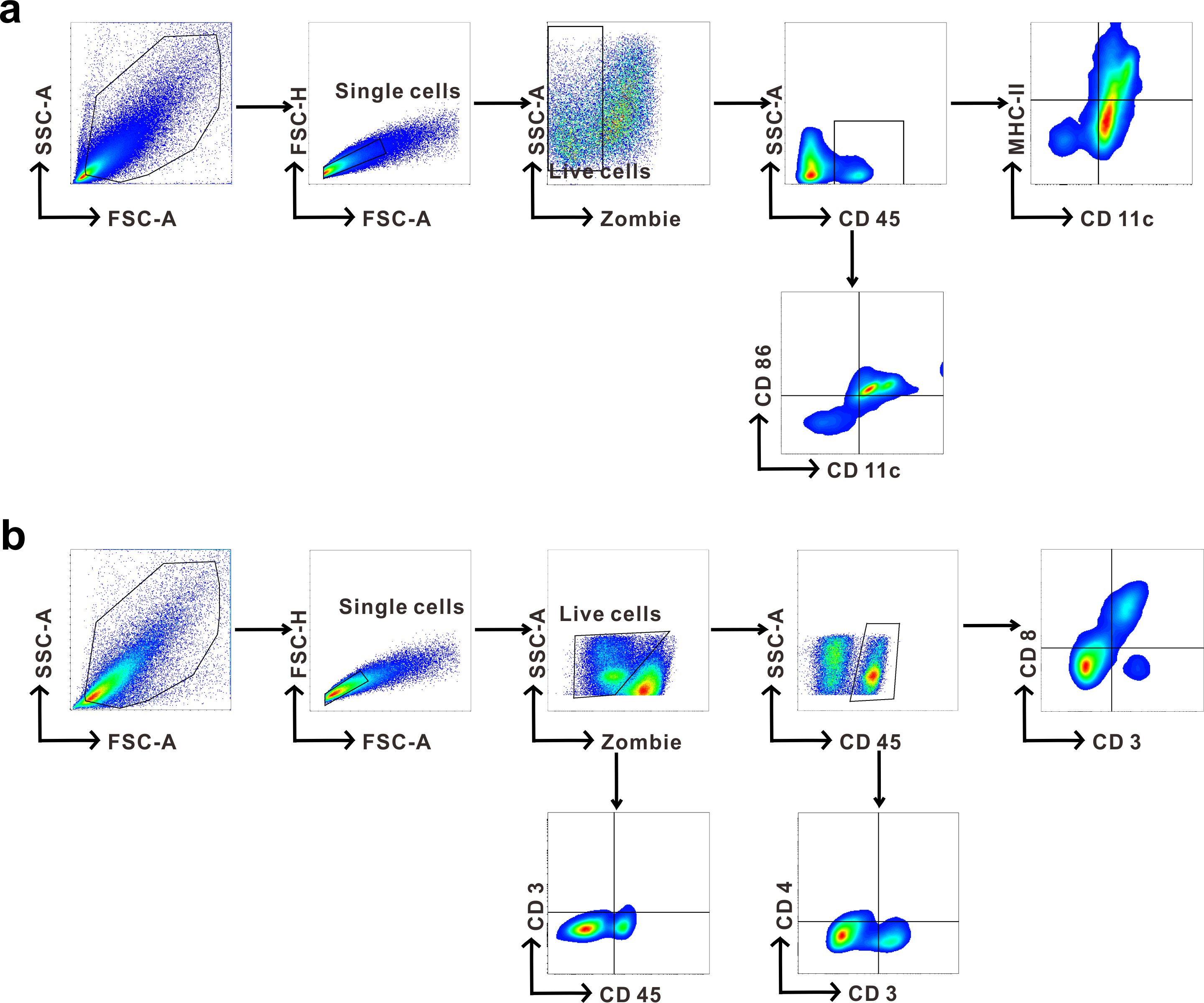


Figure S13. (a) Gating strategies for flow cytometry analysis of DCs and (b) T cells used in this study.


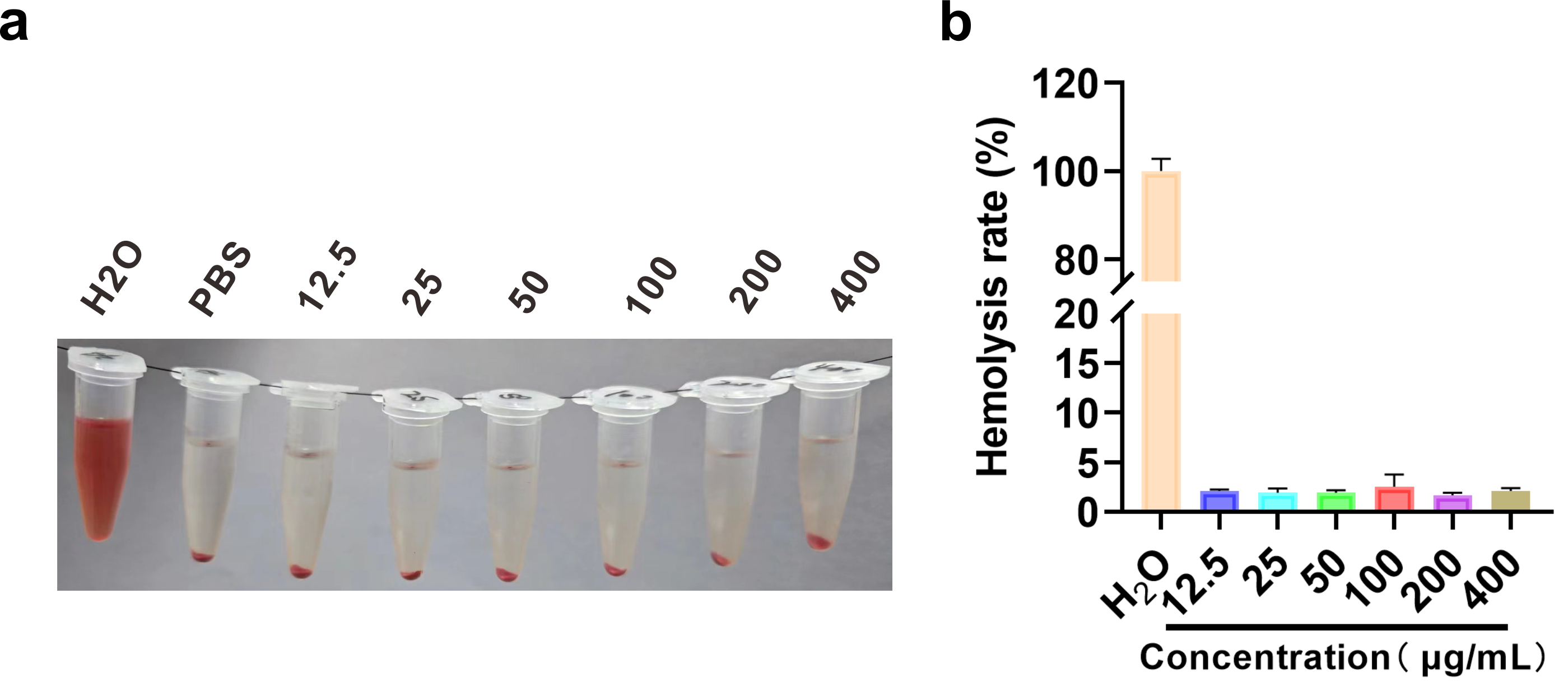


Figure S14. The hemolytic properties of Ab@Rg1/Att-ZIF was tested and statistical (n=3).
